# Defining the temporal evolution of gut dysbiosis and inflammatory responses leading to hepatocellular carcinoma in Mdr2 −/− mouse model

**DOI:** 10.1186/s12866-021-02171-9

**Published:** 2021-04-15

**Authors:** J. Behary, A. E. Raposo, N. M. L. Amorim, H. Zheng, L. Gong, E. McGovern, J. Chen, K. Liu, J. Beretov, C. Theocharous, M. T. Jackson, J. Seet-Lee, G. W. McCaughan, E. M. El-Omar, A. Zekry

**Affiliations:** 1 St George and Sutherland Clinical School, UNSW, Sydney, Australia; 2 Microbiome Research Centre, St George and Sutherland Clinical School, UNSW, Sydney, Australia; 3 Department of Gastroenterology and Hepatology, St George Hospital, Sydney, Australia; 4 Liver Injury and Cancer, Centenary Institute, University of Sydney, Sydney, Australia; 5 AW Morrow Gastroenterology and Liver Centre, Royal Prince Alfred Hospital, Sydney, Australia; 6 Department of Anatomical Pathology, St George Hospital, Sydney, Australia

**Keywords:** Hepatocellular carcinoma, Microbiome, Microbiota, Dysbiosis, Mdr2, Inflammatory response, Intrahepatic inflammation, Cirrhosis, Hepatocarcinogenesis, Lipopolysaccharide

## Abstract

**Background:**

Emerging evidence implicates the gut microbiome in liver inflammation and hepatocellular carcinoma (HCC) development. We aimed to characterize the temporal evolution of gut dysbiosis, in relation to the phenotype of systemic and hepatic inflammatory responses leading to HCC development. In the present study, Mdr2 −/− mice were used as a model of inflammation-based HCC. Gut microbiome composition and function, in addition to serum LPS, serum cytokines/chemokines and intrahepatic inflammatory genes were measured throughout the course of liver injury until HCC development.

**Results:**

Early stages of liver injury, inflammation and cirrhosis, were characterized by dysbiosis. Microbiome functional pathways pertaining to gut barrier dysfunction were enriched during the initial phase of liver inflammation and cirrhosis, whilst those supporting lipopolysaccharide (LPS) biosynthesis increased as cirrhosis and HCC ensued. In parallel, serum LPS progressively increased during the course of liver injury, corresponding to a shift towards a systemic Th1/Th17 proinflammatory phenotype. Alongside, the intrahepatic inflammatory gene profile transitioned from a proinflammatory phenotype in the initial phases of liver injury to an immunosuppressed one in HCC. In established HCC, a switch in microbiome function from carbohydrate to amino acid metabolism occurred.

**Conclusion:**

In Mdr2 −/− mice, dysbiosis precedes HCC development, with temporal evolution of microbiome function to support gut barrier dysfunction, LPS biosynthesis, and redirection of energy source utilization. A corresponding shift in systemic and intrahepatic inflammatory responses occurred supporting HCC development. These findings support the notion that gut based therapeutic interventions could be beneficial early in the course of liver disease to halt HCC development.

**Supplementary Information:**

The online version contains supplementary material available at 10.1186/s12866-021-02171-9.

## Background

Hepatocellular carcinoma (HCC) is the third leading cause of cancer related death. HCC arises in the setting of chronic liver disease with risk factors such as viral hepatitis, alcoholic liver disease, and non-alcoholic fatty liver disease [1]. These risk factors elicit a persistent intrahepatic inflammatory response, resulting in fibrosis, cirrhosis, and eventually HCC [1]. The gut microbiome has been identified as a crucial player in many chronic inflammatory conditions including chronic liver diseases [2, 3]. Various liver disorders such as alcoholic liver disease, non-alcoholic liver disease and HCC, have been associated with an altered microbiome [4–6]. Alteration in gut microbiome composition, termed ‘*dysbiosis*’, has been implicated in promoting intrahepatic inflammation and hepatocarcinogenesis through various immune mechanisms [2, 3, 5–7]. As such, gut based interventions to manipulate the microbiome are emerging as an attractive target to prevent the onset and progression of liver disease [3]. To develop these strategies, it is crucial to first understand the temporal changes of the gut microbiome in relation to the onset of systemic and intrahepatic inflammatory responses, and the sequential evolution of liver injury from inflammation to liver cirrhosis and HCC. Longitudinal studies that unveil the dynamics of interactions between the microbiome composition and function with systemic and intrahepatic inflammatory responses could define the optimal time point for gut based therapeutic interventions to prevent the progression of chronic liver disease and hepatocarcinogenesis. Clearly, animal models simulating the progression of liver injury in humans (from the onset of inflammation to cirrhosis to HCC development) are ideal for such longitudinal studies.

Multidrug resistance gene 2 knockout mice (Mdr2 −/−) provide a model of inflammation-associated HCC, and effectively capture the phases of liver injury (inflammation to cirrhosis) that is seen in humans, leading to HCC formation [8, 9]. Mdr2 −/− mice lack the liver-specific P-glycoprotein responsible for phosphatidylcholine transport across the bile canalicular membrane. This results in bile regurgitation into the portal tracts, causing inflammation, cirrhosis and eventuating in HCC development in 100% of Mdr2 −/− mice [8, 9]. Furthermore, intrahepatic gene expression profiling of Mdr2 −/− mice has shown that they exhibit many dysregulated HCC-associated genes and pathways as seen in humans [8, 9].

Therefore, the aim of this study was to examine longitudinal changes in the microbiome and its function in relation to the systemic and intrahepatic inflammatory responses in the Mdr2 −/− mouse model of HCC.

## Results

### Histology of liver injury and HCC

**Fig. 1:**
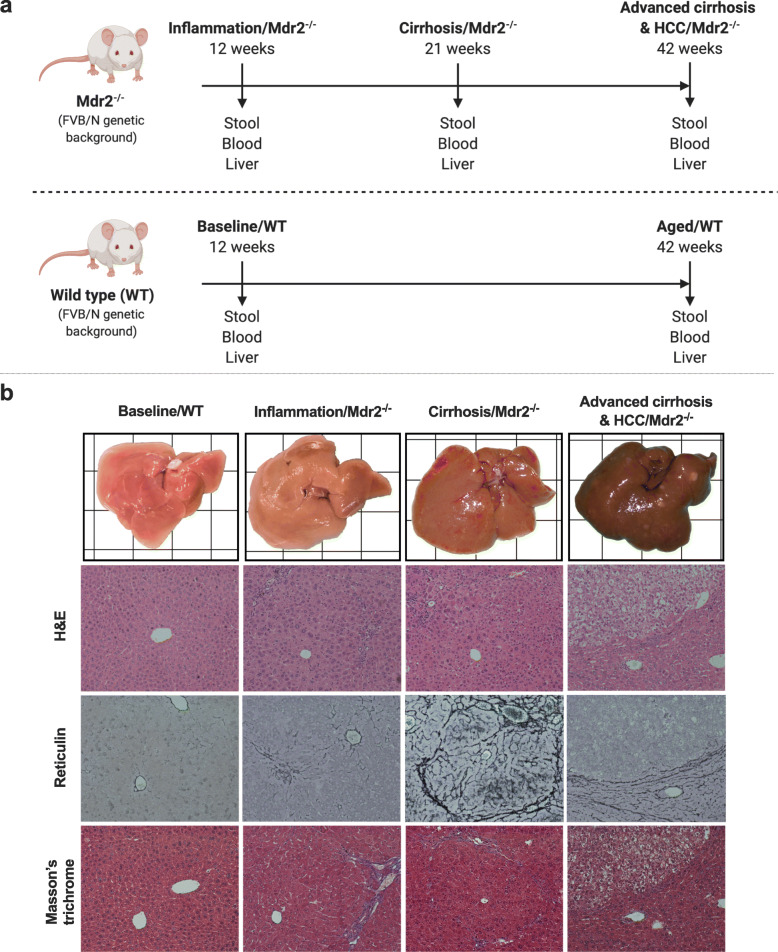
Multidrug resistant 2 knock out (Mdr2 −/−) mice: a model of inflammation mediated hepatocarcinogenesis. **a** Diagram showing time points of sample collection (stool, blood and liver tissue) of Mdr2−/− mice and respective wild type (WT) mice. Samples were collected from Mdr2−/− mice at time points reflecting liver inflammation/Mdr2−/− (12 weeks, *n* = 6), liver cirrhosis/Mdr2−/− (21 weeks, *n* = 9) and hepatocellular carcinoma (HCC)/Mdr2−/− (42 weeks, *n* = 10) after birth. Samples were collected from WT mice at 12 weeks (baseline/WT, *n* = 6) and 42 weeks (aged/WT, *n* = 6) after birth to control for effect of ageing. **b** Representative macroscopic images and histology of liver tissue from WT mice at 12 weeks (baseline/WT, *n* = 6), and Mdr2 −/− mice at 12 weeks (inflammation/Mdr2−/−, *n* = 6), 21 weeks (cirrhosis/Mdr2−/−, *n* = 9) and 42 weeks (HCC/Mdr2−/−, *n* = 10) timepoints, stained with Hematoxylin & Eosin, Reticulin and Masson’s Trichrome. Macroscopic images are to scale on background grid (10 mm × 10 mm) and histology taken at 20x magnification. Panel (**a**) was created with Biorender.com

To follow the development of liver injury, liver tissue from Mdr2 −/− mice were studied at 12, 21 and 42 weeks after birth (Fig. 1a). To control for effect of ageing, WT mice were studied at 12 weeks (baseline/WT) and 42 weeks (aged/WT) (Fig. 1a). H&E, Reticulin and Masson’s trichrome stained liver sections confirmed normal liver at baseline/WT, portal inflammation at 12 weeks, cirrhosis at 21 weeks and advanced cirrhosis with presence of HCC at 42 weeks (Fig. 1b). As expected, 100% of Mdr2 −/− mice had macroscopically visible tumours at 42 weeks (Fig. 1b) with a mean number of 17.5 (± 3.9) visible tumours that measured 3 to 19 mm in diameter. Normal liver was seen at 42 weeks in WT mice (aged/WT) (histology not shown).

### Microbiome clustering occurs with stage of disease

The fecal microbiome of all Mdr2 −/− and WT mice was profiled by 16S rRNA gene amplicon sequencing. Illumina sequencing produced a total of 5,460,928 sequences, with an average of 116,062 sequences per sample, post-quality filtering.

**Fig. 2:**
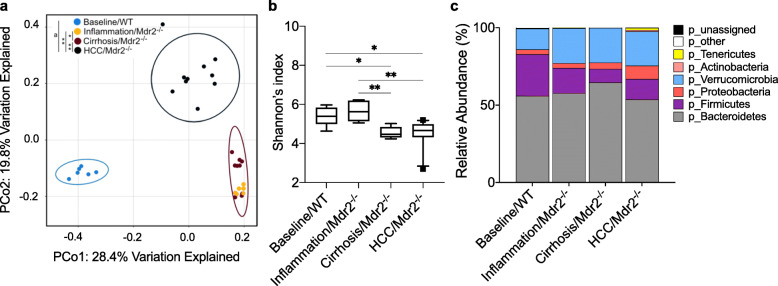
The microbiome clusters with stage of liver disease in Mdr2 −/− mice. Microbiome diversity and taxonomy at each stage of liver injury/disease (baseline/WT, *n* = 6; liver inflammation/Mdr2−/−, *n* = 6; liver cirrhosis/Mdr2−/−, *n* = 9 and hepatocellular carcinoma (HCC)/Mdr2−/−, *n* = 10) showing differences in (**a**) β-diversity by Bray–Curtis dissimilarity as displayed in the Principal Coordinates Analysis (PCoA) (**b**) α-diversity by Shannon index, box plots indicate median (middle line), 25th, 75th percentile (box) and 10th and 90th percentile (whiskers) as well as outliers (single points) and (**c**) relative taxonomic abundance at the phylum level. Permutational multivariate analysis of variance (PERMANOVA) was performed to visualize the phylogenetic distance between groups (**a**). Differences in Shannon’s index (**b**) and relative abundance (**c**) were assessed by Kruskal-Wallis for overall comparison and Dunn’s test for 2 group comparison with Benjamini-Hochberg multiple test correction, * *P* < 0.05, ** *P* < 0.01, ^a^ *P* < 0.05 in baseline/WT mice compared to all other groups. Detailed data is shown in Supporting Table 1, Additional File 2

Clustering of microbiome communities occurred with stages of liver injury and HCC in Mdr2 −/− mice. Significant separation of microbial communities occurred between all stages of progressive liver injury and with HCC development as assessed by Bray–Curtis dissimilarity matrix, shown in Principal Coordinate Analysis (PCoA) plot (*P* < 0.001) (Fig. 2a). The baseline/WT and aged/WT microbiome were clustered together and were distinctly separate from Mdr2 −/− mice, thus confirming that separation of microbial communities in Mdr2 −/− mice was a result of liver injury and HCC development rather than ageing effect (Supporting Fig. 1a, Additional File 1).

Alpha diversity as calculated by Shannon’s index was significantly reduced in liver cirrhosis (*P* = 0.038) and HCC (*P* = 0.022) when compared to baseline/WT mice (Fig. 2b). Additionally, Shannon’s index was reduced in liver cirrhosis (*P* = 0.004) and HCC (*P* = 0.002) compared to the inflammation timepoint; however, there was no difference between cirrhosis and HCC (Fig. 2b). Other indices of alpha diversity (including observed OTUs) also changed across the spectrum of liver injury, but no consistent pattern emerged (Supporting Fig. 2, Additional File 1). Importantly, no change in alpha-diversity was seen in WT mice with ageing (baseline/WT vs aged/WT mice), confirming that reduced alpha-diversity observed in Mdr2−/− mice was a result of liver injury and HCC development rather than ageing effect (Supporting Fig. 2, Additional File 1).

### Microbial composition shifts with progressive liver injury and HCC development

Sequences were classified into six phyla, accounting for 99.8% of total phyla level abundance (Fig. 2c). A clear difference in community structures at the phylum level was seen based on stage of liver injury and HCC development. Four of six measured phyla were significantly different between the different stages. Firmicutes were measured at a lower abundance during inflammation (*P* = 0.015), cirrhosis (*P* < 0.0001) and HCC (*P* = 0.0004), when compared to the baseline/WT (Fig. 2c and Supporting Fig. 3, Additional File 1). The composition of the microbiome in the inflammation stage was not significantly different from the cirrhosis stage, apart from an increase in relative abundance of Bacteroidetes during the cirrhosis time point (*P* = 0.004) (Fig. 2c and Supporting Fig. 3, Additional File 1). The HCC disease stage was characterised by an increase in the relative abundance of Tenericutes (*P* = 0.009) and Actinobacteria (*P* = 0.004) compared to the cirrhosis stage (Fig. 2c and Supporting Fig. 3, Additional File 1). No difference between phyla was observed in WT mice with ageing (baseline/WT vs aged/WT mice) (Supporting Fig. 1b, Additional File 1).

**Fig. 3:**
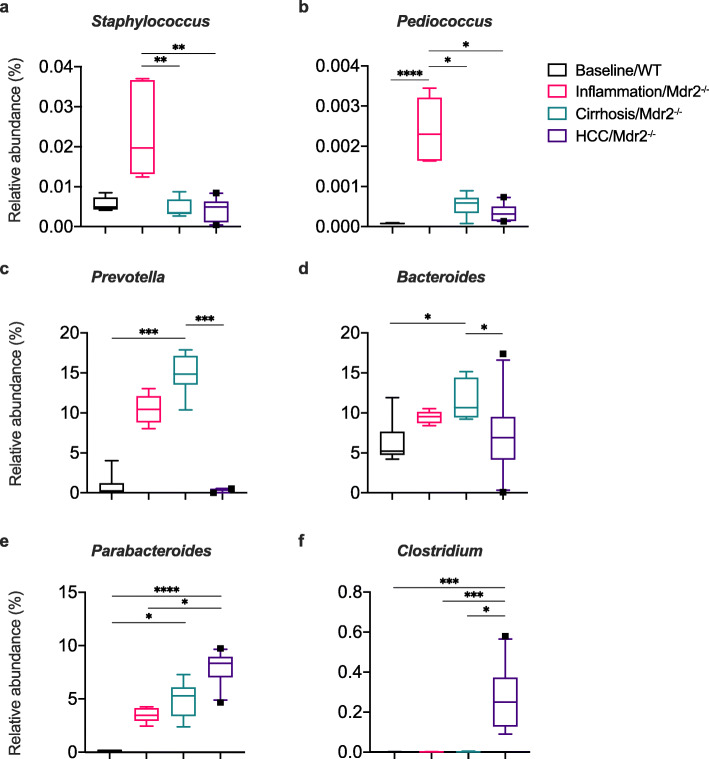
Microbiome taxonomy at genus level show key taxa enriched at the various stages of liver disease. Taxonomy (genus level) demonstrating relative abundance of enriched taxa at various stages of liver injury/disease (baseline/WT, *n* = 6; liver inflammation/Mdr2−/−, *n* = 6; liver cirrhosis/Mdr2−/−, *n* = 9 and hepatocellular carcinoma (HCC)/Mdr2−/−, *n* = 10); box plots indicate median (middle line), 25th, 75th percentile (box) and 10th and 90th percentile (whiskers) as well as outliers (single points). Difference in relative abundance were assessed by Kruskal-Wallis for overall comparison and Dunn’s test for 2 group comparison with Benjamini-Hochberg multiple test correction * *P* < 0.05; ** *P* < 0.01; *** *P* < 0.001 and **** *P* < 0.0001

At genus level, key taxa were enriched at the various stages of injury and HCC. During liver inflammation, there was an increase in *Staphylococcus* compared to cirrhosis (*P* = 0.005) and HCC time points (*P* = 0.002) (Fig. 3a). Also, an increase in *Pediococcus* was seen in inflammation compared to all other time points (all *P* < 0.0001) (Fig. 3b). In liver cirrhosis, an increase in *Prevotella* was seen compared to baseline/WT (*P* = 0.0001) and HCC (*P* = 0.0003) (Fig. 3c). Additionally, in liver cirrhosis, *Bacteroides* was enriched compared to baseline/WT (*P* = 0.024) and HCC (*P* = 0.028) (Fig. 3d). Importantly, *Parabacteroides* became more enriched with progressive liver injury/disease, with peak relative abundance occurring in HCC compared to baseline/WT (*P* < 0.001) and inflammation (*P* = 0.016) but not cirrhosis time points (Fig. 3e). *Clostridium* emerged in HCC time point, being significantly enriched compared to all other time points (all *P* < 0.05) (Fig. 3f). Detailed data is shown in Supporting Table 1, Additional File 2.

### Microbial functional capacity shifts with progressive liver injury and HCC development

**Fig. 4:**
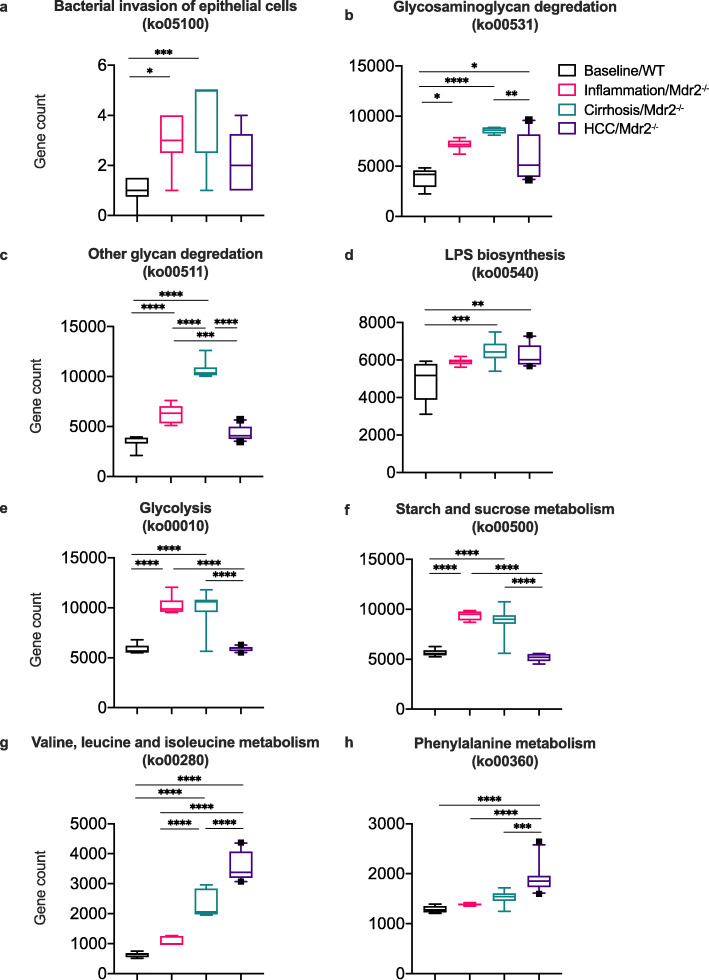
Predicted function of microbiome at the various stages of liver disease. Predicted microbial function assessed by KEGG annotation demonstrating a shift in microbial function with progression of liver injury/disease (baseline/WT, *n* = 6; liver inflammation/Mdr2−/−, *n* = 6; liver cirrhosis/Mdr2−/−, *n* = 9 and hepatocellular carcinoma (HCC)/Mdr2−/−, *n* = 10). Data presented as relative gene count (%); box plots indicate median (middle line), 25th, 75th percentile (box) and 10th and 90th percentile (whiskers) as well as outliers (single points). Differences in relative abundance were assessed by Kruskal-Wallis for overall comparison and Dunn’s test for 2 group comparison with Benjamini-Hochberg multiple test correction. *P < 0.05; ** *P* < 0.01; *** *P* < 0.001 and **** *P* < 0.0001

A range of functional pathways pertaining to the microbiome were found to be significantly increased in each of the phases of liver injury/disease in Mdr2 −/− mice. The time point of inflammation and cirrhosis were enriched with microbiome pathways related to gut barrier dysfunction, namely bacterial invasion of epithelial cells and glycosaminoglycan degradation compared to baseline/WT (all *P* < 0.05) (Fig. 4a-b and Supporting Fig. 4, Additional File 1). Functional pathways related to lipopolysaccharide (LPS) biosynthesis were elevated in cirrhosis (*P* = 0.001) and HCC (*P* = 0.003) compared to baseline/WT (Fig. 4d and Supporting Fig. 4, Additional File 1).

With respect to metabolic pathways, a theme reflecting the rearrangement of the cellular energy source was evident. Compared to baseline/WT, during liver inflammation and cirrhosis, there was an increase in microbial functional pathways related to metabolism of carbohydrates (glycolysis and starch/sucrose metabolism), which then significantly declined from cirrhosis to HCC (all *P* < 0.0001) (Fig. 4e-f and Supporting Fig. 4, Additional File 1). In contrast, functional pathways related to amino acid degradation (valine, leucine, isoleucine and phenylalanine metabolism) increased at the HCC time point compared to all other time points (all *P* < 0.0001) (Fig. 4g-h and Supporting Fig. 4, Additional File 1). Detailed data is shown in Supporting Table 2, Additional File 2.

### Progressive liver injury and HCC development is associated with increased serum LPS and a shift toward a Th1/Th17 proinflammatory cytokine milieu

**Fig. 5:**
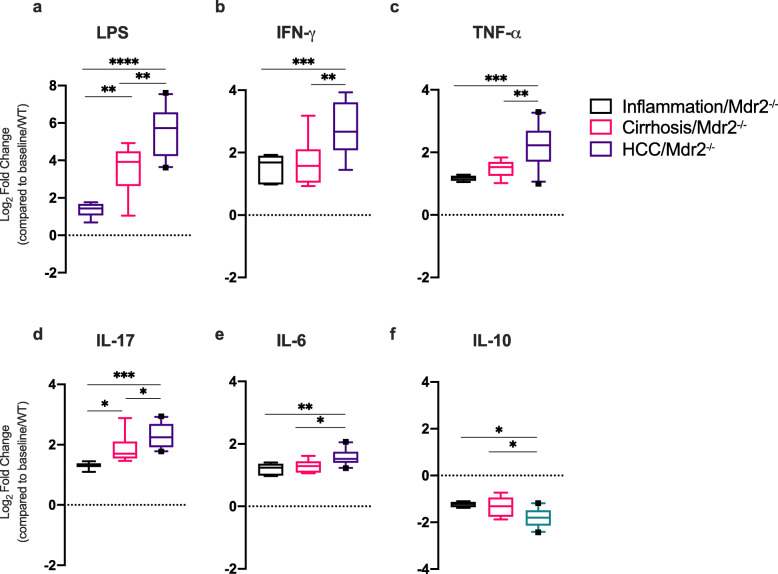
Progressive liver disease is associated with a rise in serum LPS and development of a Th1/Th17 proinflammatory cytokine milieu. Serum LPS and cytokine levels demonstrating development of a proinflammatory systemic response with liver injury/disease progression (liver inflammation/Mdr2−/−, *n* = 6; liver cirrhosis/Mdr2−/−, *n* = 9 and hepatocellular carcinoma (HCC)/Mdr2−/−, *n* = 10). The mean LPS/cytokine level of the baseline/WT time point (*n* = 6) is represented as the dashed horizontal line, from which log2 fold change (Log2FC) is calculated; a value above the dashed line represents an increased cytokine concentration compared to baseline/WT, and a value below the dashed line represents decreased cytokine concentration compared to baseline/WT. Box plots indicate median (middle line), 25th, 75th percentile (box) and 10th and 90th percentile (whiskers) as well as outliers (single points). *P* values are calculated by one-way ANOVA for overall comparison and Tukey’s test for 2 group comparison. *P < 0.05; ** *P* < 0.01; *** *P* < 0.001 and **** *P* < 0.0001. Detailed data is shown in Supporting Fig. 5, Additional File 1 and Supporting Table 3, Additional File 2

In the serum, LPS levels increased with progressive liver injury and peaked with development of HCC in Mdr2 −/− mice. Serum LPS levels were higher at the HCC time point compared to both inflammation and cirrhosis timepoints (*P* < 0.0001 and *P* = 0.002, respectively) (Fig. 5a). In parallel, a shift toward a Th1/Th17 proinflammatory cytokine milieu was seen with HCC development. The HCC timepoint was characterized by a significant increase in interferon-gamma (IFN-γ), tumour necrosis factor alpha (TNF-α), IL-6 and IL-17 compared to inflammation and cirrhosis timepoints (all *P* < 0.05) (Fig. 5b-e). Other proinflammatory cytokines followed a similar pattern (Supporting Fig. 5, Additional File 1). The converse was seen with the anti-inflammatory cytokine, IL-10, which was significantly reduced in the serum at the HCC timepoint compared to inflammation and cirrhosis timepoints (*P* = 0.022 and *P* = 0.032, respectively) (Fig. 5f). Detailed data is shown in Supporting Table 3, Additional File 2.

### Transcriptional profiling of mouse liver tissue demonstrates alterations in intrahepatic inflammatory responses across the spectrum of liver injury to HCC

**Fig. 6:**
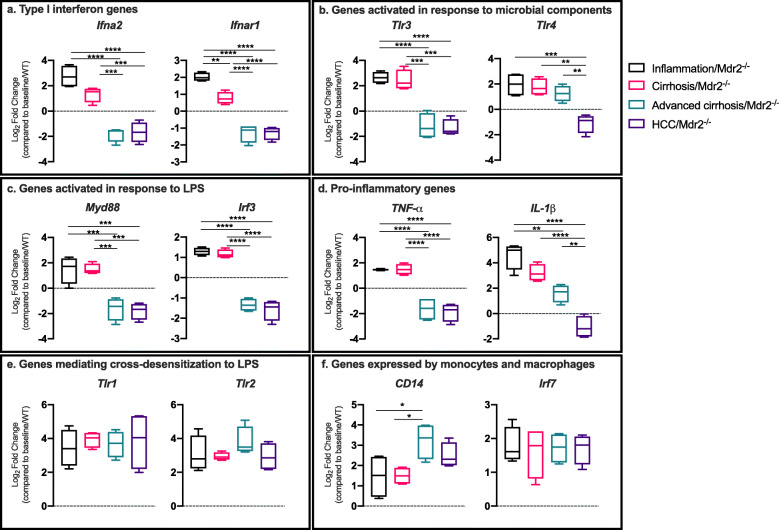
Changes in the intrahepatic inflammatory response with progression of liver disease demonstrating a shift from a proinflammatory to an immunosuppressed profile as HCC ensues. Fold change from baseline of key intrahepatic genes with progressive liver injury/disease in Mdr2 −/− mice (liver inflammation/Mdr2−/−, *n* = 4; liver cirrhosis/Mdr2−/−, *n* = 4 and advanced cirrhosis/hepatocellular carcinoma (HCC)/Mdr2−/−, *n* = 4). Advanced cirrhosis represents the peritumoral tissue at 42 weeks, whilst HCC represents the tumor tissue proper at 42 weeks. The mean gene expression level of the baseline/WT timepoint (*n* = 4) is represented as the dashed horizontal line, from which log_2_ fold change (Log_2_FC) is calculated; a value above the dashed line represents an increased gene expression compared to baseline/WT, and a value below the dashed line represents decreased gene expression compared to baseline/WT; box plots indicate median (middle line), 25th, 75th percentile (box) and 10th and 90th percentile (whiskers) as well as outliers (single points). *P* values are calculated by one-way ANOVA for overall comparison and Tukey’s test for 2 group comparison. *P < 0.05; ** *P* < 0.01; *** *P* < 0.001 and **** *P* < 0.0001. Detailed data is shown in Supporting Fig. 6, Additional File 1 and Supporting Table 4, Additional File 2

Evaluation of the expression of genes throughout the spectrum of liver injury in Mdr2 −/− mice (*n* = 4 per group) showed a significant increase in intrahepatic inflammatory responses during inflammation and cirrhosis time points, which was followed by immunosuppression with the development of HCC (Supporting Fig. 6, Additional File 1). Upregulation of type I (Ifn) encoding genes (*Ifna2* and *Ifnar1*), important in first line defense against microbial invasion and loss of immune tolerance [10] were seen in inflammation and cirrhosis stages compared to all other timepoints (all *P* < 0.01) (Fig. 6a). Genes activated in response to microbial components *Tlr3* and *Tlr4* followed a similar pattern, elevated during inflammation and cirrhosis stages compared to the HCC timepoint (all *P* < 0.01) in Mdr2 −/− livers (Fig. 6b). In parallel, genes activated by LPS including myeloid differentiation factor 88 (*Myd88*) and interferon regulatory factor 3 (*Irf3*) were upregulated in inflammation and cirrhosis compared to advanced cirrhosis and HCC (all *P* < 0.001) (Fig. 6c). Several proinflammatory mediators, for example *TNF-α* and *IL-1β* (Fig. 6d) and *CCR5*, followed the same pattern in Mdr2 −/− livers (all *P* < 0.01).

In contrast to the predominant proinflammatory milieu detected at the inflammation and cirrhosis time points, a blunted inflammatory response prevailed in advanced cirrhosis and HCC. When comparing advanced cirrhosis with HCC time points, a further reduction in several proinflammatory cytokines including *Tlr4* (Fig. 6b) and *IL-1β* (Fig. 6d) was seen (*P* = 0.0037 and *P* = 0.0032, respectively). There was, however, ongoing overexpression of *Tlr1*, *Tlr2* (Fig. 6e) and *Tlr6* genes throughout all time points including HCC compared to baseline/WT. *Tlr1*, *Tlr2* and *Tlr6,* are known to recognize lipopeptides and to mediate cross-desensitization to LPS [11, 12] and their expression coincided with the emergence of microbial functional pathways relating to bacterial invasion of the gut barrier and increased LPS biosynthesis pathways in addition to increased serum LPS levels.

Furthermore, genes typically expressed by monocytes/macrophages, *CD14* and *Irf7*, were overexpressed throughout the course of liver injury with further elevation of *CD14* in advanced cirrhosis compared to both inflammation and cirrhosis time points (*P* = 0.037 and *P* = 0.041, respectively) (Fig. 6f). Detailed data is shown in Supporting Table 4, Additional File 2.

### Changes in intrahepatic inflammatory responses correlate with changes in gut microbial composition and serum LPS level

To identify relationships between gut microbiome composition and the intrahepatic immune response, correlation analysis was performed between microbial abundance (genus level) and expression of intrahepatic genes across all stages of liver injury and HCC in Mdr2 −/− mice. Additionally, given its integral importance to both microbial and intrahepatic inflammatory responses, serum LPS levels were included in our correlation analysis.

**Fig. 7:**
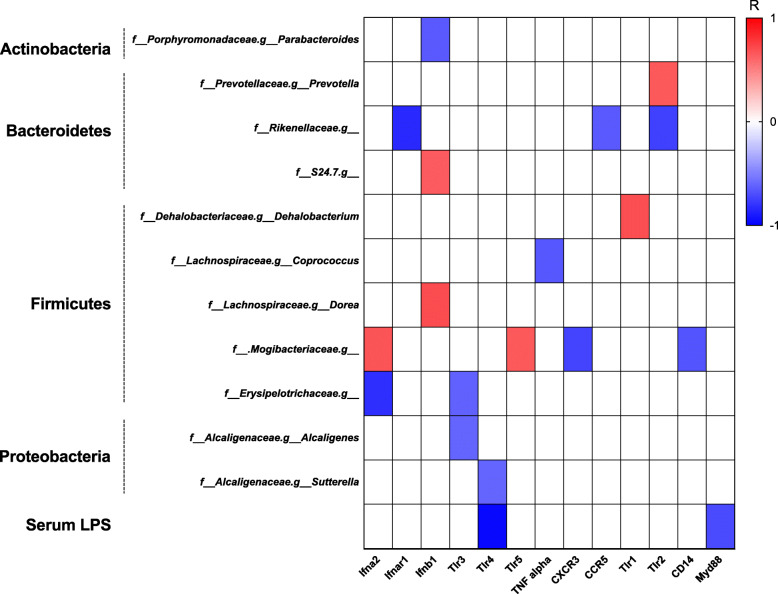
Several intrahepatic genes correlate with LPS and enriched taxa in Mdr2 −/− mice. Spearman’s correlation between microbial abundance (genus level) and serum LPS with intrahepatic genes. Color legend represents correlation coefficient (R). All strong and significant correlations (*R* > 0.65 or *R* < − 0.65, *P* < 0.05 after Benjamini-Hochberg multiple test correction) are shown

Following correction for multiple testing (FDR > 0.25), Spearman’s correlation analysis revealed a number of host intrahepatic genes that covaried with gut microbial abundance in Mdr2 −/− mice (Fig. 7). Overall, changes in the expression levels of key genes had both positive and negative correlations with changes in abundance of various genera primarily from the phyla Bacteroidetes, Firmicutes and Proteobacteria. To this effect, *Prevotella* (phylum Bacteroidetes) enriched in cirrhosis was positively correlated with *Tlr2*, a gene elevated in cirrhotic Mdr2−/− livers and important in LPS cross-desensitization (R = 0.65, *P* = 0.041) (Fig. 7). Enrichment of *Parabacteroides* (phylum Actinobacteria) seen at the HCC time point was negatively correlated with the inflammatory gene *Ifnb1* (R = − 0.65, *P* = 0.038) (Fig. 7).

Serum LPS levels, shown to increase throughout the time course of liver disease (Fig. 5a), were found to be negatively correlated with *Tlr4* (R = − 0.97, *P* = 0.033) and *Myd88* (R = − 0.71, *P* = 0.041) (Fig. 7); genes which are well established to be involved in macrophage mediated LPS immunotolerance.

## Discussion

**Fig. 8:**
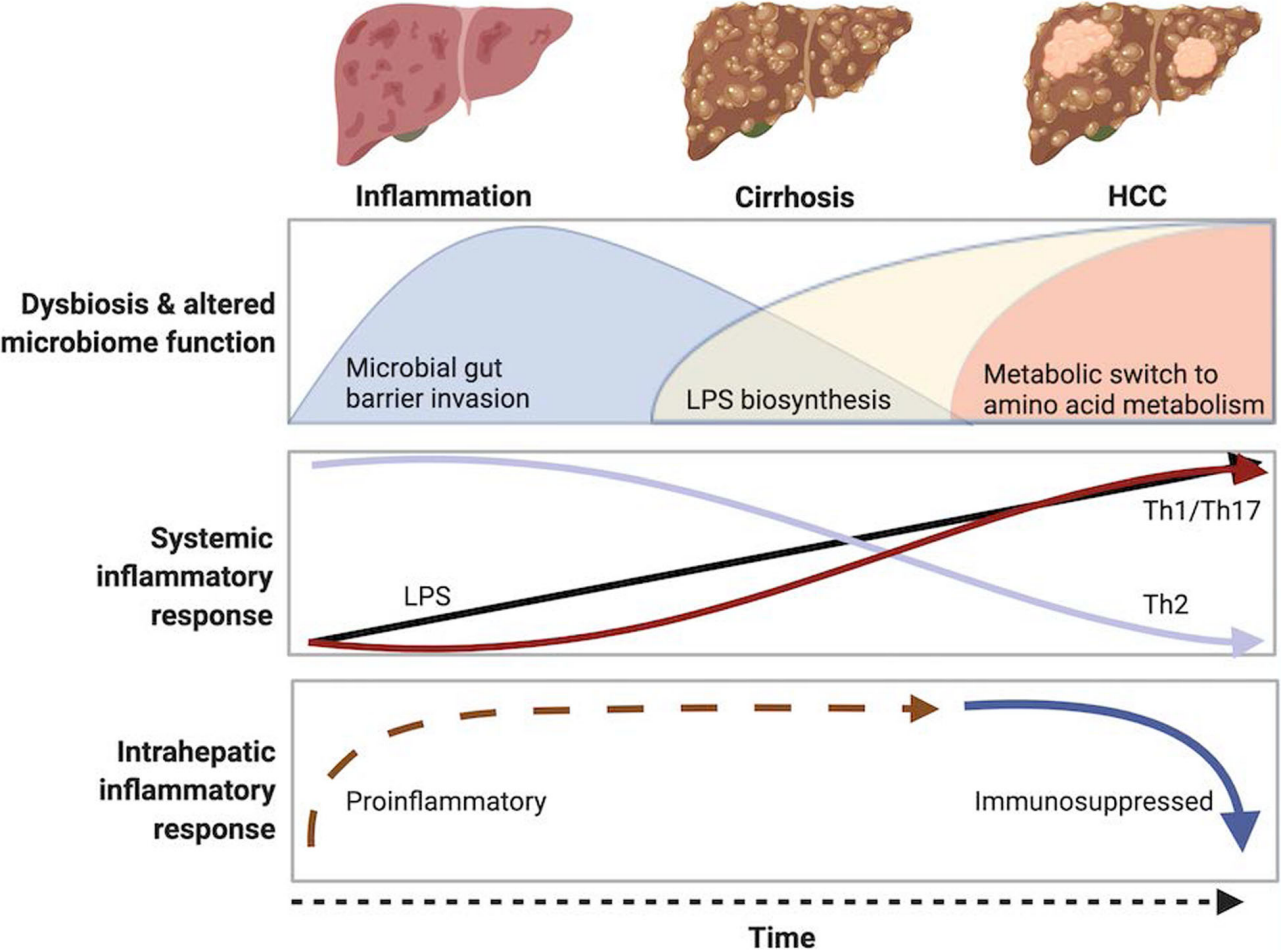
The temporal evolution of the gut microbiome, its functionality and inflammatory responses in Mdr2 −/− model of HCC. Schema summarizing key findings. The Mdr2 −/− mouse models exemplifies the process of inflammation mediated hepatocarcinogenesis as seen in humans. Dysbiosis and altered function of the microbiome occur with progression of liver injury/disease and hepatocarcinogenesis. A shift in functionality is seen with changes in intestinal barrier function early, followed by increased LPS biosynthesis and altered metabolic pathways with progressive liver disease. These changes occur in parallel with increased serum LPS levels, and a shift towards a Th1/Th17 cytokine milieu. In HCC there is a switch in microbiota function from carbohydrate to amino acid metabolism which may support hepatocarcinogenesis through direct and/or indirect mechanisms. The findings suggest that gut-based interventions should be timed early in the disease process to halt hepatocarcinogenesis. Figure was created with Biorender.com

The gut-liver-immune axis is the key mechanism by which the gut microbiome promotes liver disease and hepatocarcinogenesis [6, 7]. Given the increasing burden of HCC globally, a better understanding of the pathophysiological connections between gut dysbiosis and the hepatic injury is crucial for the development of gut based therapeutic interventions to treat chronic liver diseases or to at least, prevent its progression to HCC [3]. In this study, we tracked longitudinally in a mouse model of HCC, the temporal evolution of the gut microbiome, in relation to systemic and intrahepatic inflammatory responses, leading on to HCC formation. We made key observations: firstly, dysbiosis occurred with liver inflammation and preceded HCC development. Secondly, during the initial course of liver inflammation and early cirrhosis, composition and functional shifts in the microbiome supported gut barrier dysfunction and bacterial invasion; this corresponded to upregulation of intrahepatic genes important in providing first-line defense against microbial invasion, such as type I interferons and other proinflammatory genes. Next, with advanced cirrhosis and HCC, microbial function related to LPS biosynthesis ensued, thus supporting a progressive rise in serum LPS levels, and the emergence of a systemic Th1/Th17 proinflammatory cytokine response. As liver injury progressed to advanced cirrhosis and HCC, the heightened peripheral levels of LPS and the associated systemic inflammatory responses were contrasted with the emergence of a predominantly blunted intrahepatic inflammatory response, which is known to support HCC establishment. Thirdly, there was a switch in microbiome function from carbohydrate to amino acid metabolism, which may inadvertently promote cancer survival (Fig. 8). Finally, correlative models confirmed a link between certain bacteria taxa and serum LPS, with intrahepatic inflammatory responses cascading to HCC development.

Compositional changes in gut microbiome have been described in chronic liver disease and HCC, with typical findings of increased Bacteroidetes, reduced Firmicutes, and a rise in proinflammatory taxa such as Proteobacteria [13–16]. The association between these taxa and HCC formation is strengthened by studies that demonstrate their depletion through broad spectrum antibiotic administration results in reduction in number and size of HCC lesions in both genetic and toxin induced HCC mouse models [2, 17]. In our study, we found changes within these phyla occurred early in the course of liver injury. Gut dysbiosis has been previously shown to lead to disrupted gut barrier function, leading to increased gut bacterial translocation and initiation of a proinflammatory pattern of immune activation in the intestine as well as the periphery [18, 19]. In this study, there was evidence that gut dysbiosis related microbial function supported a breakdown of the gut barrier early in the phase of liver injury. Correspondingly we noted triggering of an intrahepatic inflammatory response against microbial “invasion” with induction of expression of proinflammatory genes, such as those encoding type I interferons, and genes such as *Tlr3* and *Tlr4*, known to possess a bacteriolytic function. The majority of these genes are known to break down the liver ‘immune tolerance’ phenotype [20, 21] resulting in increased expression of other proinflammatory genes, as was observed at the time points of liver inflammation and early cirrhosis. Hence in agreement with other groups, we noted that the initial dysbiosis related events seemed to trigger the launch of a proinflammatory response, a key initial step in responding to bacterial pathogens and in initiating liver injury [18].

With liver disease progressing to cirrhosis, it was observed that microbial function related to LPS biosynthesis ensued and corresponded to increasing serum levels of LPS with cirrhosis and HCC. The accumulation of LPS is recognised to foster a pathogenic process characterised by increased bacterial translocation, increased intestinal permeability and the promotion of proinflammatory responses in the periphery and in the liver [22]. In this setting, pattern recognition receptors, expressed in many cells, have been shown to recognise LPS, and mediate the interaction between the immune system and the gut microbiome [22, 23]. This results in promoting the release of proinflammatory cytokines TNF-α, IL-6 and IL-17, as well as activating signalling pathways involved in hepatocarcinogenesis [24]. In concordance with this published data, we detected in cirrhosis and HCC that with dysbiosis, a measurable shift occurs toward a systemic Th1/Th17 proinflammatory cytokine phenotype, characterised by increased levels of IFN- γ, TNF-α, IL-6 and IL-17 amongst others. Therefore, it appears that gut dysbiosis-dependent events, and related inflammatory responses act synergistically to support or inadvertently promote environment required for HCC development.

With further progression of liver injury to advanced cirrhosis and HCC, a distinctive occurrence with evolving gut dysbiosis, was the coexistence of a heightened systemic inflammation and a contrasting blunted intrahepatic inflammatory response (Fig. 8). This finding supports other work indicating that repeated or prolonged exposure of inflammatory cells within the liver to bacterial products, such as lipopeptides, can result in dampened immune responses to subsequent exposure to LPS through cross-desensitization [11, 12], a fundamental mechanism responsible for intrahepatic ‘immunoparalysis’ [25, 26]. Repeated LPS exposure has also been demonstrated to dampen macrophage mediated *Tlr4-Myd88* signalling pathways [25]. In support, in the current study, we observed strong negative correlations between serum LPS and intrahepatic *Tlr4* and *Myd88*, thereby in concordance with the observation that initial unrestricted proinflammatory responses to bacterial products contribute to ‘LPS immunotolerance’ and the immunosuppressed intrahepatic phenotype observed in advanced cirrhosis and HCC. It is worth noting that within this global immunosuppressed intrahepatic environment, a further reduction in key intrahepatic genes activated in response to microbial components and LPS, including *Tlr4* and *IL-1β* was seen in HCC compared to advanced cirrhosis. Taken together, findings from this work support the notion that under constant bacterial pressure, reprogramming of the immune response within the liver occurs with a switch from a predominantly proinflammatory phenotype to a predominantly immunodeficient one, thus favouring HCC development and survival [27].

Other microbiome related functional pathways which were of note were those pertaining to metabolism. Metabolic alterations characterise the carcinogenic process, to satisfy the demands of cancer cell growth, proliferation and survival. Importantly, alteration in glycolysis and metabolism have been shown to suppress inflammatory macrophage activation, and production of proinflammatory cytokines, hence promoting an immunosuppressive environment [28]. This is relevant as in HCC, as there is evidence of altered glucose metabolism, with increased utilisation of other pathways (amino acids and lipid peroxidation) to generate energy [29]. We have noted a shift in microbiome-related metabolic pathways preceding HCC development. Hence taken together, it is plausible that as liver disease advances to HCC, the shift in metabolic function of the microbiome may inadvertently support tumour formation and growth.

The current study has some limitations: Firstly, the Mdr2 −/− model, although advantageous in its ability to capture important phases of inflammation associated HCC development, is not an inducible model, thus making it difficult to ascertain whether microbiome perturbations were as a result of liver disease or vice versa. This limitation was in part mitigated by analysis of microbiota in age-matched WT mice (FVB/N) mice. Secondly, the animal model used relies on bile acid perturbations to induce HCC. Bile acids have been shown to affect the composition of the microbiome, and it could be argued that the microbial changes seen were as a result of bile acid perturbations. Measurement of bile acids and other metabolites would have provided additional insight to functional pathways and mechanisms involved with progression of liver injury and HCC development. However, the longitudinal nature of this study still enabled characterizing dynamics pertaining to shifts in microbial composition, its functionality and related intrahepatic inflammatory events as liver disease evolved to HCC.

## Conclusion

In the present study, we found during the progression of liver disease to HCC, dysbiosis ensues, with microbiome functional capacity supporting loss of intestinal integrity during liver inflammation, and increased exposure to LPS during cirrhosis and HCC development. This evolution in microbial responses corresponded to increased systemic inflammation, and the progressive establishment of a suppressed intrahepatic inflammatory state, with advanced cirrhosis and HCC. We postulate based on the findings that gut based interventions, in inflammation and/or early cirrhosis may halt the progression of liver disease to HCC.

## Methods

### Mouse model

Male multidrug resistance gene 2 (Mdr2 −/−) mice with FVB/N genetic background, along with male FVB/N mice (WT), were obtained from The Jackson Laboratory (The Jackson Laboratory, Bar Harbour, ME, United States). Mice were maintained at the Centenary Institute Animal Facility under humane and specific pathogen-free (SPF) conditions. The Animal Welfare Committee (AWC), Sydney Local Health District, approved all experimental procedures and protocols (Ethics approval: 2014/007, 2018/016). To minimise potential confounders, after 2 weeks of acclimatisation, animals were co-housed (3 to 5 per cage) according to their designated endpoints in a temperature-controlled facility (22 °C), 12:12 light dark cycle, with food (Standard Chow, Mouse Maintenance Diet, Specialty Feeds, WA, Australia) and water available ad libitum. From Mdr2 −/− mice, stool pellets, whole blood and liver tissue were collected at designated experimental endpoints; 12 weeks (inflammation/Mdr2−/−; inflammation) (*n* = 6), 22 weeks (cirrhosis/Mdr2−/−; cirrhosis) (*n* = 9) and 42 weeks (hepatocellular carcinoma (HCC)/Mdr2−/−; HCC) (*n* = 10) (Fig. 1a). At 42 weeks liver tissue was additionally obtained from peritumoral tissue (advanced cirrhosis/Mdr2−/−; advanced cirrhosis) (Fig. 1a). From WT mice, faecal samples, whole blood and liver tissue were collected at 12 weeks (baseline/WT) (*n* = 6) and 42 weeks (aged/WT) (*n* = 6) (Fig. 1a). Each mouse was treated as one experimental unit; the total number of mice was *n* = 37 with no specific inclusion or exclusion criteria applied. Stool pellets were collected aseptically by placing individual mice in a sterile container, waiting for natural defecation, and harvesting of at least 2 pellets per mouse. Whole blood and liver samples were collected immediately following carbon dioxide asphyxiation and cervical dislocation. All samples were stored at -4 °C at the time of collection until required for downstream analysis. Study investigators who allocated/conducted experiments were not blinded (as there was no treatment/intervention), however, those who performed sample analysis were blinded to study timepoints.

### DNA extraction and 16S rRNA gene sequencing

Stool samples were homogenized using Qiagen TissueLyser II (Qiagen, #85300) at 30 Hz for 5 min. Total genomic DNA was extracted from mouse stool using the PSP® Spin Stool DNA Plus Kit (Stratec, # 1038100300) as per manufacturer’s instructions. DNA quantity was measured by Qubit™ dsDNA Broad Range Assay Kit (ThermoFisher, #Q32853) and Qubit Fluorometer (Life Technologies). Library preparation and sequencing was performed using 341F and 805R primers for the V3-V4 region of the 16S rRNA gene on the Illumina MiSeq System (Illumina, #SY-410-1003) with paired 300 bp reads at the Ramaciotti Centre for Genomics (UNSW Sydney).

### Microbiome analysis

Forward and reverse reads of the 16S rRNA gene were imported to Quantitative Insights into Microbial Ecology (Qiime2) [30]. The DADA2 pipeline [31] was used for detecting and correcting Illumina amplicon sequences, removal of primers and chimeric reads, and assembly into amplicon sequence variants (ASVs) [32]. Taxonomy was assigned using a naïve Bayes classifier trained on the Greengenes v13_8 99% database [33]. Alpha diversity was presented using standard indices. Beta diversity was calculated using Bray Curtis distance. PICRUSt2 [34] was used to perform the microbial gene inference analysis on the ASVs. ASVs singletons were removed and absolute abundances were normalized using the inferred 16S rRNA gene copy counts. The predicted KEGG orthologs was obtained using default parameters. KEGG database (v77.1) provided in PICRUSt2 was used to obtain pathway-level abundances.

### Serum LPS, cytokine and chemokine analysis

Serum LPS was measured in mouse serum with Pierce™ Chromogenic Endotoxin Quanti Kit (ThermoFisher, #A39553) as per manufacturer’s instructions. In brief, 50 μL of serum and 50 μL of standards were prepared and read at density of 405 nm in a microplate reader, immediately following assay completion. Mouse Cytokine/Chemokine Convenience 26-Plex ProcartaPlex Panel (ThermoFisher, #EPXR260–26088-901) was used to measure 26 cytokines and chemokines in 25 μL mouse serum as per manufacturer’s instructions. Samples were prepared without dilution, and a total of 8 standards were used per cytokine to generate standard curves. The plate was read on MAGPIX (Luminex xMAP) and data were analysed with Bio-Plex Manager software (version 6.0).

### Liver histology

Liver tissue was fixed in 10% formaldehyde, embedded in paraffin, and cut into 5 μm-thick sections for H&E, Reticulin and Masson’s trichrome stain as per standard protocols. All sections were reviewed and scored by a single blinded expert histopathologist.

### Ribonucleic acid (RNA) extraction

Liver samples (5–20 mg) were homogenized in 700 μL QIAzol lysis reagent (Qiagen, # 79306) with Qiagen Tissuelyzer II (Qiagen, #85300) at 30 Hz for 2 min. Total RNA was extracted using miRNeasy Mini Kit (Qiagen, #217004) according to the manufacturer’s instructions. RNA quantity and quality were evaluated by Nanodrop™ 2000 UV-Vis spectrophotometer (Thermo Fisher Scientific # ND-2000).

### Complementary DNA (cDNA) synthesis

cDNA was prepared from 500 ng of extracted RNA using RT2 First Strand Kit (Qiagen, #330404) according to the manufacturer’s instructions. The final mix was submitted to the following cycling conditions: 42 °C for 15 min and 95 °C for 5 min. All cDNA was diluted 1:4 with nuclease-free water and stored at − 20 °C or used immediately for qRT-PCR.

### Quantitative real-time (RT) PCR

cDNA from livers of Mdr2 −/− and WT mice were subjected to gene expression analysis using a panel of innate and adaptive immune response markers. qRT-PCR reactions were performed according to the manufacturer’s instructions. All reagents were obtained from Qiagen. qRT-PCR was performed using RT^2^ Profiler PCR array (Qiagen, # 330231) with RT^2^ SYBR Green Master Mix (Qiagen, #330502) using 1 μL cDNA input per well. qRT-PCR was performed on CFX96 Touch™ Real-Time PCR system (Bio-Rad, #1855196) with the following cycling conditions: initial denaturation at 95 °C for 10 min followed by amplification for 40 cycles (95 °C for 15 s and 60 °C for 1 min) with a ramp rate of 1 °C/second. This was followed by a melt curve analysis from 65 °C to 95 °C at a rate of 1 °C/second. The Ct value of endogenous control genes (*actb, b2m, gapdh, gusb, hsp90ab1*) were subtracted from the corresponding Ct value for the target gene resulting in the ΔCt value which was used for relative quantification of miRNA expression using the 2^- (ΔΔCt) method.

### Statistical analysis

Differences in microbiome alpha diversity, abundance and functional pathways were assessed using non-parametric analysis; Kruskal-Wallis test followed by Dunn’s multiple comparison test with Benjamini-Hochberg multiple test correction. Principal Coordinates Analysis (PCoA) ​generated using Bray Curtis dissimilarity metric was to visualize the ​bacterial community structure of the Mdr2 −/− and WT mice. Differences between the groups were identified using permutational multivariate analysis of variance (PERMANOVA) with Benjamini-Hochberg multiple test correction. Statistical analyses for serum cytokines/chemokines and liver inflammatory markers were assessed by one-way ANOVA and post-hoc Tukey’s multiple comparisons test following log transformation. Results are presented as mean log_2_ fold change from baseline (baseline/WT). Spearman’s non-parametric correlation was performed to analyze relationships between the microbiome, serum LPS and expression of intrahepatic inflammatory genes. Results are presented as correlation coefficient (R). *P* values of < 0.05 were considered statistically significant. Statistical analyses were performed with R v3.5.3 and figures were generated by Prism v8.2.1 (GraphPad Software, Inc.). No mice were excluded in any of the data analysis.

## Supplementary Information

**Additional file 1: Supporting Fig. 1.** Microbiome analysis confirms no significant differences in microbiome profiles in wild type (WT) mice as a result of ageing. **Supporting Fig. 2.** Indices of alpha diversity of the gut microbiome are stable with ageing in wild type (WT) mice but altered with progressive liver injury in Mdr2−/− mice. **Supporting Fig. 3.** Microbiome taxonomy at phylum level show key taxa enriched at the various stages of disease. **Supporting Fig. 4.** Microbiome functional signatures shift with progression of liver disease. **Supporting Fig. 5.** Fold regulation of cytokines and chemokines in serum across the spectrum of liver disease. **Supporting Fig. 6.** Fold regulation of expression of innate and adaptive genes within the liver across the spectrum of liver disease.

**Additional file 2: Supporting Table 1.** Taxonomic composition of the microbiome at the phylum and genus level in stool of Mdr2 −/− mice with progressive livery injury and HCC. **Supporting Table 2.** Predicted function of the microbiome at the phylum and genus level in stool of Mdr2 −/− mice with progressive livery injury and HCC. **Supporting Table 3.** LPS and cytokine/chemokine levels in the serum of Mdr2 −/− mice with progressive livery injury and HCC. **Supporting Table 4.** Intrahepatic gene expression in Mdr2 −/− mice with progressive livery injury and HCC.

## Data Availability

All data generated or analyzed during this study are included in this published article and its supplementary information files.

## Abbreviations

HCC: Hepatocellular carcinoma
LPS: Lipopolysaccharide
Mdr2: Multidrug resistance gene 2
OTU: Observed taxonomic units
IL: Interleukin
CD: Cluster of differentiation
IFN: Interferon
*Ifna*: Interferon alpha
*Ifnb*: Interferon beta
*Ifnar*: Interferon alpha receptor
*Irf*: Interferon regulatory factor
*Myd88*: Myeloid differentiation factor 88
TNF: Tumor necrosis factor
*Tlr*: Toll-like receptor
