# Supplementary material for: Defining the temporal evolution of gut dysbiosis and inflammatory responses leading to hepatocellular carcinoma in Mdr2 −/− mouse model
Source: BMC Microbiol. 2021 Apr 15;21:113. doi: 10.1186/s12866-021-02171-9 (PMC8048083; doi:10.1186/s12866-021-02171-9)
Supplement: Supplementary file 1 — Additional file 1: Supporting Fig. 1. Microbiome analysis confirms no significant differences in microbiome profiles in wild type (WT) mice as a result of ageing. Supporting Fig. 2. Indices of alpha diversity of the gut microbiome are stable with ageing in wild type (WT) mice but altered with progressive liver injury in Mdr2−/− mice. Supporting Fig. 3. Microbiome taxonomy at phylum level show key taxa enriched at the various stages of disease. Supporting Fig. 4. Microbiome functional signatures shift with progression of liver disease. Supporting Fig. 5. Fold regulation of cytokines and chemokines in serum across the spectrum of liver disease. Supporting Fig. 6. Fold regulation of expression of innate and adaptive genes within the liver across the spectrum of liver disease. [file 12866_2021_2171_MOESM1_ESM.pdf]

ADDITIONAL FILE 1

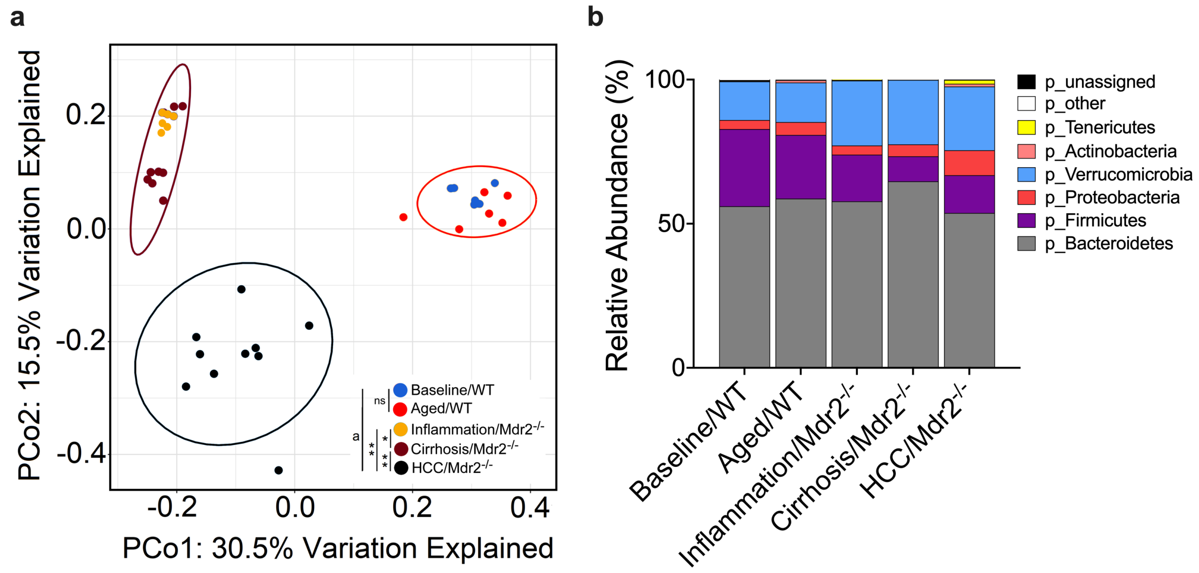

Supporting Fig 1: Microbiome analysis confirms no significant differences in microbiome profiles in wild type (WT) mice as a result of ageing.

(a) Microbiota diversity (β-diversity) by Bray–Curtis dissimilarity as displayed in the Principal Coordinates Analysis (PCoA) and (b) microbiota taxonomy at the phylum level are not significantly different in wild type (WT) mice at 12 weeks of age (baseline/WT, n=6) and 42 weeks (aged/WT, n=6). However, microbiota diversity (β-diversity) (a) and taxonomy (b) diverge with progression of liver injury in Mdr2-/- mice. Permutational multivariate analysis of variance (PERMANOVA) was performed to visualize the phylogenetic distance between groups (a); * $P < 0.05$; ** $P < 0.01$, [a] $P < 0.05$ in WT mice compared to all other groups, [ns] not statistically significant. Differences in microbiota taxonomy between groups are shown in detail in Fig 2, Fig 3, Supporting Fig 2 and Supporting Table 1, Additional File 2.

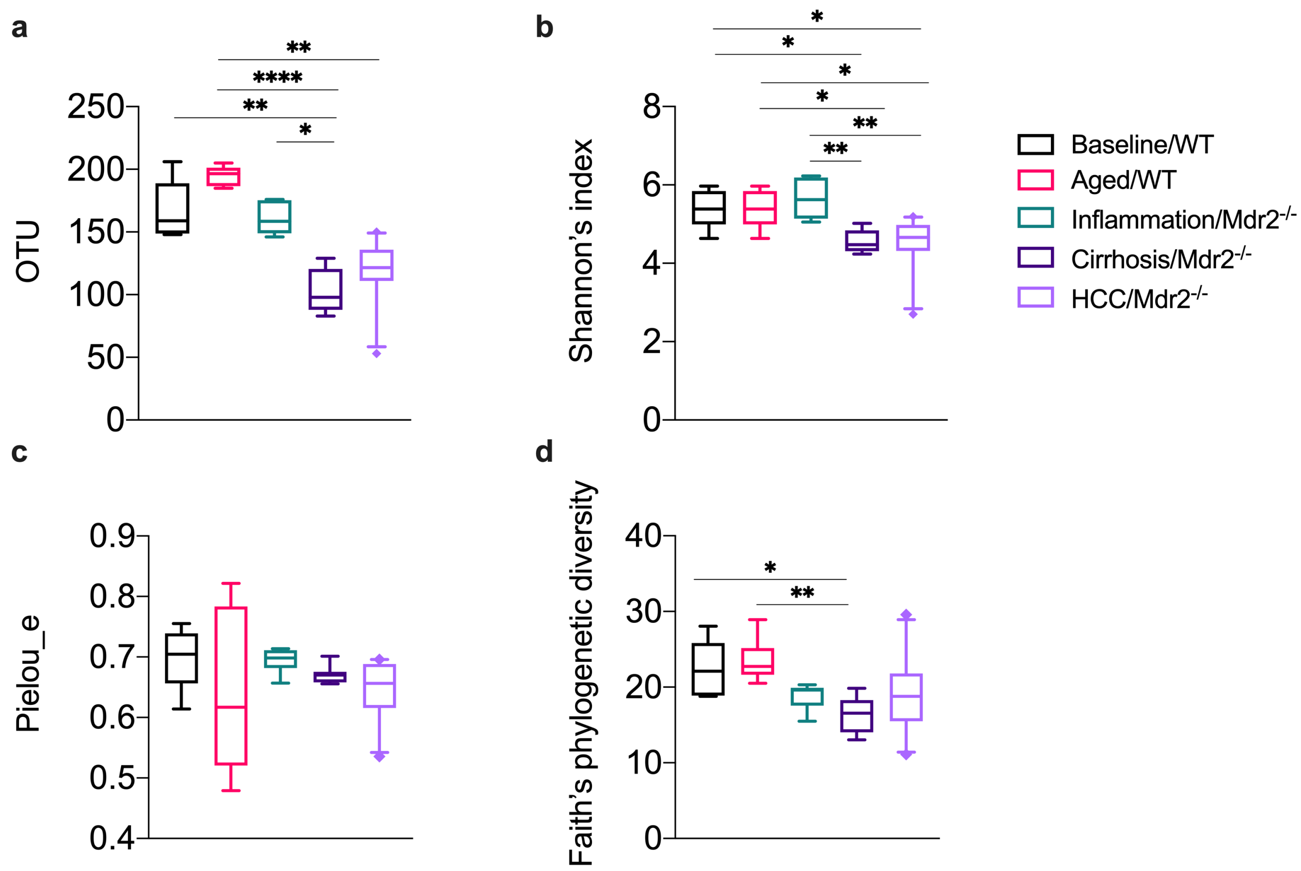

Supporting Fig 2: Indices of alpha diversity of the gut microbiome are stable with ageing in wild type (WT) mice but altered with progressive liver injury in Mdr2-/- mice.

Alpha-diversity indices, namely (a) observed OTUs, (b) Shannon's index, (c) Pileou_e and (d) Faith Phylogenic Diversity in wild type (WT) mice and with progressive liver injury in Mdr2 -/- mice. Baseline/WT (n=6), Aged/WT (n=6), liver inflammation/Mdr2-/- (n=6), liver cirrhosis/Mdr2-/- (n=9) and hepatocellular carcinoma (HCC)/Mdr2-/- (n=10). Box plots indicate median (middle line), 25th, 75th percentile (box) and 10th and 90th percentile (whiskers) as well as outliers (single points). Differences in alpha diversity indices were assessed by Kruskal-Wallis for overall comparison and Dunn's test for 2 group comparison with Benjamini-Hochberg multiple test correction. *$P < 0.05$; ** $P < 0.01$; **** $P < 0.0001$.

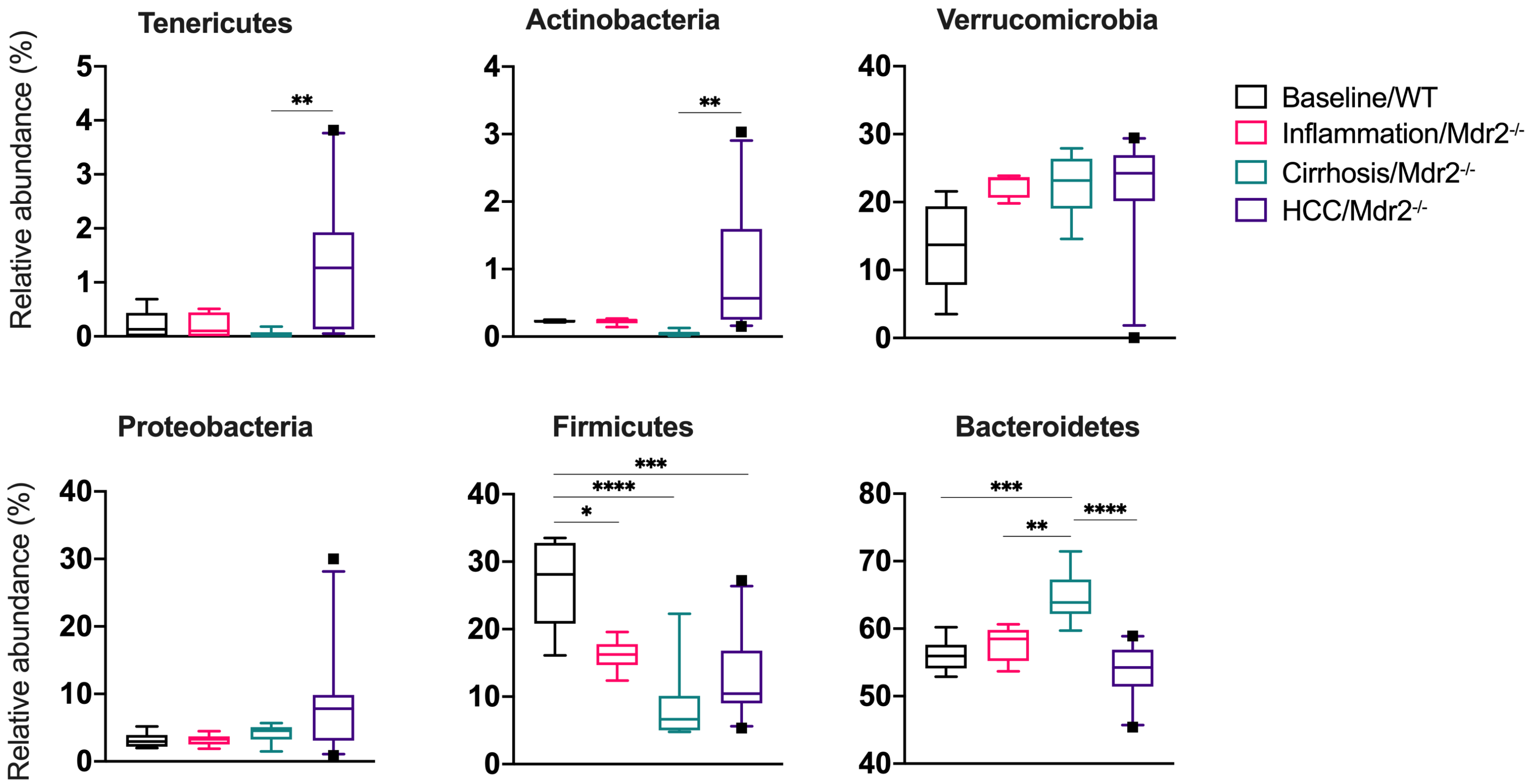

Supporting Fig 3: Microbiome taxonomy at phylum level show key taxa enriched at the various stages of disease.
Taxonomic annotation of the gut microbiome at the phylum level with progressive livery injury/disease (baseline/WT, n=6; liver inflammation/Mdr2-/- , n=6; liver cirrhosis/Mdr2-/-, n=9 and hepatocellular carcinoma (HCC)/Mdr2-/-, n=10); box plots indicate median (middle line), 25th, 75th percentile (box) and 10th and 90th percentile (whiskers) as well as outliers (single points). Differences in relative abundance were assessed by Kruskal-Wallis for overall comparison and Dunn's test for 2 group comparison with Benjamini-Hochberg multiple test correction. *P < 0.05; ** P < 0.01; *** P < 0.001 and **** P < 0.0001. Additional detail is shown in Supporting Table 1, Additional File 2.

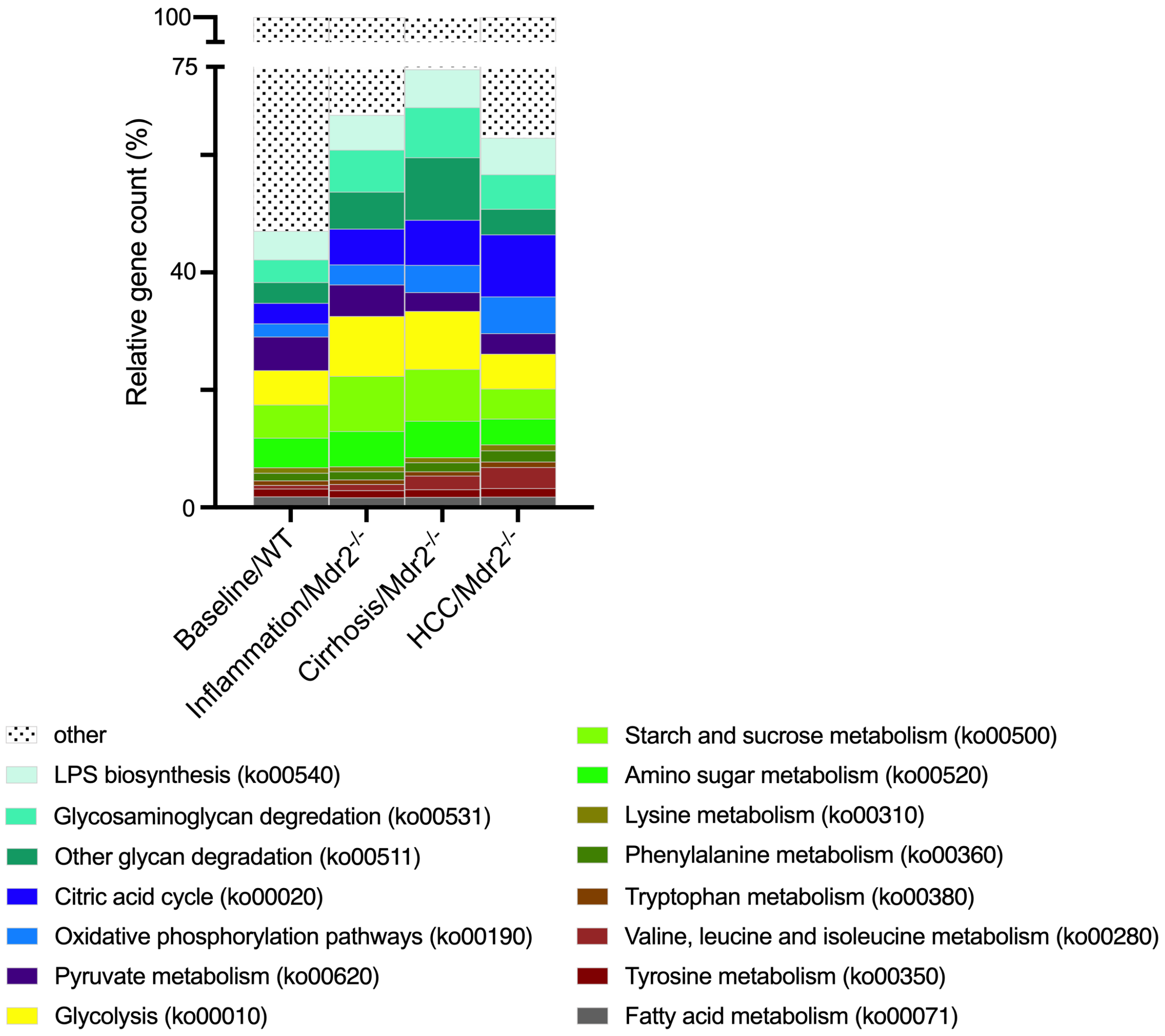

Supporting Fig 4: Microbiome functional signatures shift with progression of liver disease.

Predicted microbial function assessed by KEGG annotation demonstrating a shift in microbial function with progression of liver injury/disease (baseline/WT, n=6; liver inflammation/Mdr2-/-, n=6; liver cirrhosis/Mdr2-/-, n=9 and hepatocellular carcinoma (HCC)/Mdr2-/-, n=10). Data presented as mean relative gene count (%) of 15 abundant and relevant functions. Detailed data including comparisons between groups are shown in Fig 4 and Supporting Table 2, Additional File 2.

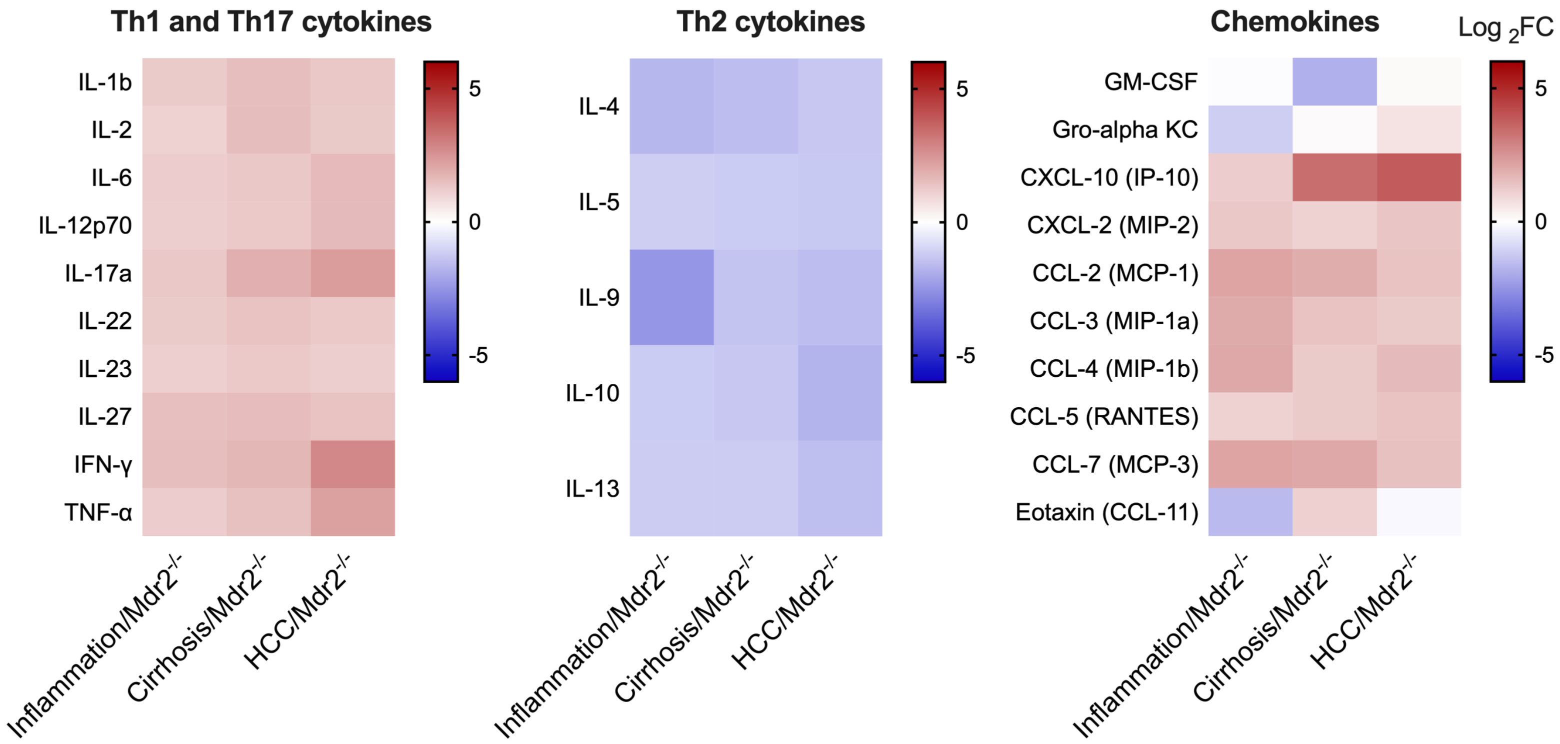

Supporting Fig 5: Fold regulation of cytokines and chemokines in serum across the spectrum of liver disease

Heat map demonstrating changes in fold regulation of Th1, Th17, Th2 cytokines and chemokines with progressive liver injury/disease in Mdr2 -/- mice (liver inflammation/Mdr2-/-, n=6; liver cirrhosis/Mdr2-/-, n=9 and hepatocellular carcinoma (HCC)/Mdr2-/-, n=10). Color legend represents the $\log_2$ fold change (Log_2FC) calculated from the mean cytokine level of the baseline/WT timepoint (n=6). Detailed data is shown in Supporting Table 3, Additional File 2.

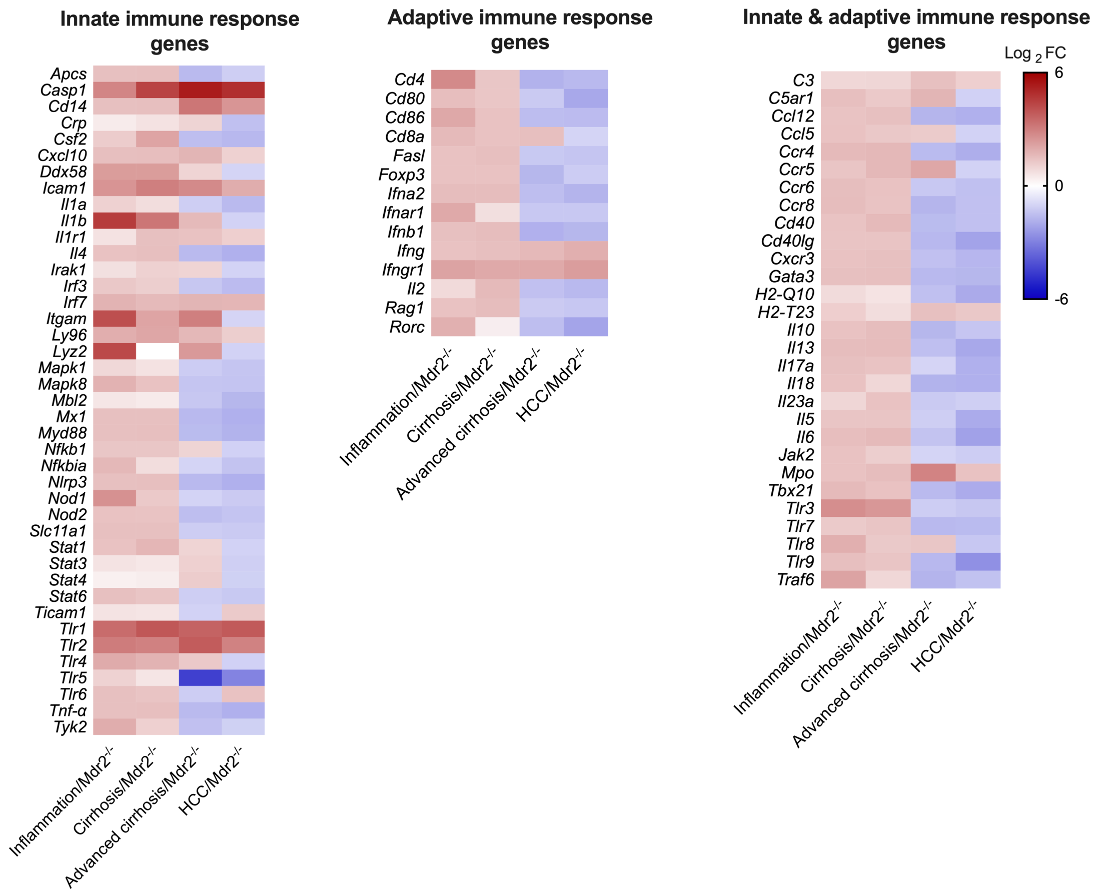

Supporting Fig 6: Fold regulation of expression of innate and adaptive genes within the liver across the spectrum of liver disease

Heat map demonstrating changes in fold regulation of expression of innate and adaptive immune response genes with progressive liver injury/disease in Mdr2 -/- mice (liver inflammation/Mdr2-/-, n=4; liver cirrhosis/Mdr2-/-, n=4 and advanced cirrhosis/hepatocellular carcinoma (advanced cirrhosis/Mdr2-/- and HCC/Mdr2-/-), n=4). Advanced cirrhosis represents peritumoral tissue at 42 weeks, whilst HCC represents the tumor tissue proper at 42 weeks in Mdr2 -/- mice. Color legend represents the $\log_2$ fold change (Log_2FC) calculated from the mean gene expression level of the baseline/WT timepoint (n=4). Detailed data is shown in Supporting Table 4, Additional File 2.
