# Supplementary material for: Defining the temporal evolution of gut dysbiosis and inflammatory responses leading to hepatocellular carcinoma in Mdr2 −/− mouse model
Source: BMC Microbiol. 2021 Apr 15;21:113. doi: 10.1186/s12866-021-02171-9 (PMC8048083; doi:10.1186/s12866-021-02171-9)
Supplement: Supplementary file 2 — Additional file 2: Supporting Table 1. Taxonomic composition of the microbiome at the phylum and genus level in stool of Mdr2 −/− mice with progressive livery injury and HCC. Supporting Table 2. Predicted function of the microbiome at the phylum and genus level in stool of Mdr2 −/− mice with progressive livery injury and HCC. Supporting Table 3. LPS and cytokine/chemokine levels in the serum of Mdr2 −/− mice with progressive livery injury and HCC. Supporting Table 4. Intrahepatic gene expression in Mdr2 −/− mice with progressive livery injury and HCC. [file 12866_2021_2171_MOESM2_ESM.pdf]

ADDITIONAL FILE 2

Supporting Table 1. Taxonomic composition of the microbiome at the phylum and genus level in stool of Mdr2 -/- mice with progressive livery injury and HCC
Data is shown as mean ± standard deviation (SD) of relative abundance (%). *P* values calculated by one-way Kruskal-Wallis (KW) test for 3 group comparison and Dunn’s post hoc test for 2 group comparisons. *P* < 0.05 considered statistically significant.

Taxonomy (relative abundance, %)	Baseline/WT Mean (±SD)	Inflammation/Mdr2-/- Mean (±SD)	Cirrhosis/Mdr2-/- Mean (±SD)	HCC/Mdr2-/- Mean (±SD)	*P*-value (KW test)	*P*-value Baseline/WT vs Inflammation/Mdr2-/-	*P*-value Baseline/WT vs Cirrhosis/Mdr2-/-	*P*-value Baseline/WT vs HCC/Mdr2-/-	*P*-value Inflammation/Mdr2-/- vs Cirrhosis/Mdr2-/-	*P*-value Inflammation/Mdr2-/- vs HCC/Mdr2-/-	*P*-value Cirrhosis/Mdr2-/- vs HCC/Mdr2-/-
**Phylum**											
p_Tenericutes	2.167E-01(±2.655E-01)	1.883E-01(±2.208E-01)	3.667E-02(±7.314E-02)	1.294E+00(±1.332E+00)	0.007	>0.9999	0.972	0.059	0.983	0.051	0.009
p_Actinobacteria	2.310E-01(±1.619E-02)	2.280E-01(±4.479E-02)	5.167E-02(±3.570E-02)	9.866E-01(±9.289E-01)	0.004	>0.9999	0.920	0.051	0.924	0.050	0.004
p_Verrucomicrobia	1.340E+01(±6.974E+00)	2.251E+01(±1.702E+00)	2.235E+01(±4.318E+00)	2.212E+01(±8.473E+00)	0.036	0.078	0.052	0.054	>0.9999	0.999	1.000
p_Proteobacteria	3.135E+00(±1.160E+00)	3.183E+00(±8.634E-01)	4.109E+00(±1.319E+00)	8.636E+00(±8.195E+00)	0.076	>0.9999	0.981	0.147	0.983	0.152	0.198
p_Firmicutes	2.680E+01(±6.603E+00)	1.618E+01(±2.395E+00)	8.690E+00(±5.523E+00)	1.310E+01(±6.439E+00)	<0.0001	0.015	<0.0001	0.0004	0.081	0.720	0.344
p_Bacteroidetes	5.605E+01(±2.478E+00)	5.776E+01(±2.578E+00)	6.471E+01(±3.574E+00)	5.372E+01(±4.275E+00)	<0.0001	0.831	0.000	0.580	0.004	0.140	<0.0001
**Genus level**											
o__Bacillales;f__Staphylococcaceae;g__Staphylococcus	5.617E-03(±1.720E-03)	2.313E-02(±1.139E-02)	4.773E-03(±2.357E-03)	4.071E-03(±2.956E-03)	0.002	0.087	>0.9999	>0.9999	0.005	0.002	>0.9999
o__Lactobacillales;f__Lactobacillaceae;g__Pediococcus	7.588E-05(±1.194E-05)	2.408E-03(±8.101E-04)	5.372E-04(±2.615E-04)	3.545E-04(±2.141E-04)	<0.0001	<0.0001	0.147	0.534	<0.0001	<0.0001	0.749
o__Bacteroidales;f__Prevotellaceae;g__Prevotella	8.011E-01(±1.568E+00)	1.046E+01(±1.965E+00)	1.503E+01(±2.415E+00)	3.632E-01(±1.699E-01)	<0.0001	0.055	0.0001	>0.9999	0.985	0.179	0.0003
o__Bacteroidales;f__Bacteroidaceae;g__Bacteroides	6.288E+00(±2.832E+00)	9.477E+00(±8.275E-01)	1.173E+01(±2.529E+00)	7.033E+00(±4.687E+00)	0.008	0.555	0.024	>0.9999	>0.9999	0.850	0.028
o__Clostridiales;f__Clostridiaceae;g__Clostridium	1.633E-03(±2.805E-04)	1.650E-03(±2.881E-04)	2.556E-03(±1.258E-03)	2.670E-01(±1.601E-01)	<0.0001	>0.9999	>0.9999	0.001	>0.9999	0.001	0.025
o__Bacteroidales;f__Porphyromonadaceae;g__Parabacteroides	1.333E-01(±7.421E-02)	3.483E+00(±6.725E-01)	4.910E+00(±1.623E+00)	7.933E+00(±1.426E+00)	<0.0001	0.718	0.044	<0.0001	>0.9999	0.016	0.141

Supporting Table 2. Predicted function of the microbiome at the phylum and genus level in stool of Mdr2 -/- mice with progressive livery injury and HCC

Data is shown as mean ± standard deviation (SD) of gene count. *P* values calculated by one-way Kruskal-Wallis (KW) test for 3 group comparison and Dunn's post hoc test for 2 group comparisons. *P* < 0.05 considered statistically significant.

Predicted function (gene count)	Baseline/WT Mean (±SD)	Inflammation/Mdr2-/- Mean (±SD)	Cirrhosis/Mdr2-/- Mean (±SD)	HCC/Mdr2-/- Mean (±SD)	*P*-value (KW test)	*P*-value Baseline/WT vs Inflammation/Mdr2-/-	*P*-value Baseline/WT vs Cirrhosis/Mdr2-/-	*P*-value Baseline/WT vs HCC/Mdr2-/-	*P*-value Inflammation/Mdr2-/- vs Cirrhosis/Mdr2-/-	*P*-value Inflammation/Mdr2-/- vs HCC/Mdr2-/-	*P*-value Cirrhosis/Mdr2-/- vs HCC/Mdr2-/-
LPS biosynthesis (ko00540)	4883.833(±1074.847)	5911.333(±186.430)	6459.222(±603.920)	6225.000(±566.962)	0.001	0.055	0.001	0.003	0.409	0.794	0.866
Bacterial invasion of epithelial cells (ko05100)	1.000(±0.548)	3.000(±1.095)	3.778(±1.563)	2.300(±1.160)	0.001	0.037	0.001	0.182	0.617	0.677	0.058
Glycosaminoglycan degredation (ko00531)	3851.167(±967.810)	7161.667(±537.254)	8557.667(±298.443)	5889.400(±2230.597)	<0.0001	0.002	<0.0001	0.039	0.245	0.304	0.001
Other glycan degredation (ko00511)	3542.167(±705.740)	6275.417(±945.927)	10648.500(±838.751)	4335.550(±758.904)	<0.0001	<0.0001	<0.0001	0.255	<0.0001	0.001	<0.0001
Citric acid cycle (ko00020)	3496.500(±340.457)	6090.333(±279.642)	7672.556(±385.772)	10585.600(±1861.576)	<0.0001	0.002	<0.0001	<0.0001	0.054	<0.0001	<0.0001
Oxidative phosphorylation pathways (ko00620)	2239.667(±81.884)	3451.167(±130.667)	4618.444(±386.108)	6236.200(±600.774)	<0.0001	0.0001	<0.0001	<0.0001	<0.0001	<0.0001	<0.0001
Pyruvate metabolism (ko00620)	5717.833(±255.888)	5326.167(±73.385)	3202.778(±215.069)	3527.100(±143.501)	<0.0001	0.005	<0.0001	<0.0001	<0.0001	<0.0001	0.004
Glycolysis (ko00010)	5862.167(±496.877)	10204.833(±942.916)	9905.333(±1753.324)	5903.600(±255.637)	<0.0001	<0.0001	<0.0001	1.000	0.951	<0.0001	<0.0001
Starch and sucrose metabolism (ko00500)	5653.833(±348.567)	9376.833(±457.887)	8806.444(±1375.013)	5135.400(±400.629)	<0.0001	<0.0001	<0.0001	0.619	0.560	<0.0001	<0.0001
Amino sugar metabolism (ko00520)	5015.833(±553.455)	6038.167(±105.302)	6198.778(±244.884)	4393.600(±1140.123)	<0.0001	0.086	0.020	0.350	0.973	0.001	<0.0001
Phenylalanine metabolism (ko00360)	1287.500(±73.519)	1287.500(±73.519)	1526.000(±132.872)	1904.800(±283.361)	<0.0001	0.785	0.085	<0.0001	0.474	<0.0001	0.001
Trytophan metabolism (ko00380)	763.000(±54.750)	767.333(±37.972)	748.000(±36.633)	928.500(±87.923)	<0.0001	0.999	0.967	<0.0001	0.933	0.000	<0.0001
Valine, leucine and isoleucine metabolism (ko00280)	633.333(±81.461)	1073.167(±146.243)	2299.444(±439.315)	3583.900(±479.399)	<0.0001	0.198	<0.0001	<0.0001	<0.0001	<0.0001	<0.0001
Tyrosine metabolism (ko00350)	1324.167(±213.777)	1199.333(±50.532)	1306.333(±50.988)	1454.400(±106.182)	0.002	0.267	0.991	0.156	0.319	0.001	0.046
Fatty acid metabolism (ko00071)	1854.000(±110.002)	1686.167(±71.965)	1775.444(±53.156)	1822.600(±167.739)	0.079	0.081	0.579	0.952	0.473	0.128	0.812

Supporting Table 3. LPS and cytokine/chemokine levels in the serum of Mdr2 -/- mice with progressive livery injury and HCC

Data is shown as Log2 fold change (log2FC) from baseline/WT time point. *P* values calculated by one-way ANOVA test for 3 group comparison and Tukey's post hoc test for 2 group comparisons. $P < 0.05$ considered statistically significant.

Serum LPS and cytokines/chemokines (Log2 Fold Change from Baseline/WT)	Inflammation/Mdr2-/- Mean (±SD)	Cirrhosis/Mdr2-/- Mean (±SD)	HCC/Mdr2-/- Mean (±SD)	*P*-value (one-way ANOVA)	*P*-value Inflammation/Mdr2-/- vs Cirrhosis/Mdr2-/-	*P*-value Inflammation/Mdr2-/- vs HCC/Mdr2-/-	*P*-value Cirrhosis/Mdr2-/- vs HCC/Mdr2-/-
**LPS**	1.361(±0.383)	3.500(±1.230)	5.602(±1.297)	<0.0001	0.004	<0.0001	0.002
**Th1 and Th17**							
IL-1b	1.229(±0.124)	1.513(±0.270)	1.300(±0.195)	0.037	0.049	0.798	0.099
IL-2	1.068(±0.044)	1.532(±0.471)	1.265(±0.260)	0.040	0.036	0.490	0.205
IL-6	1.198(±0.181)	1.282(±0.199)	1.578(±0.262)	0.005	0.759	0.009	0.023
IL-12p70	1.141(±0.147)	1.263(±0.355)	1.608(±0.379)	0.023	0.766	0.031	0.082
IL-17a	1.316(±0.118)	1.865(±0.448)	2.308(±0.410)	0.0002	0.031	0.0001	0.048
IL-22	1.238(±0.297)	1.431(±0.366)	1.262(±0.275)	0.411	0.489	0.988	0.486
IL-23	1.129(±0.042)	1.262(±0.202)	1.154(±0.115)	0.162	0.208	0.940	0.252
IL-27	1.488(±0.047)	1.553(±0.436)	1.412(±0.268)	0.627	0.919	0.887	0.600
IFN-γ	1.526(±0.435)	1.682(±0.739)	2.795(±0.843)	0.003	0.914	0.008	0.008
TNF-α	1.179(±0.087)	1.467(±0.268)	2.223(±0.673)	0.000	0.475	0.001	0.005
**Th2**							
IL-4	-1.738(±0.328)	-1.554(±0.316)	-1.328(±0.236)	0.034	0.461	0.030	0.227
IL-5	-1.165(±0.034)	-1.227(±0.213)	-1.272(±0.219)	0.562	0.813	0.533	0.866
IL-9	-2.476(±0.466)	-1.411(±0.236)	-1.557(±0.528)	0.0002	0.0003	0.001	0.742
IL-10	-1.233(±0.113)	-1.324(±0.414)	-1.777(±0.403)	0.011	0.883	0.022	0.032
IL-13	-1.212(±0.188)	-1.204(±0.149)	-1.551(±0.532)	0.090	0.999	0.190	0.117
**Chemokines**							
GM-CSF	-0.057(±1.569)	-1.871(±1.234)	0.137(±1.327)	0.009	0.047	0.959	0.010
Gro-alpha KC	-1.156(±0.110)	0.116(±1.369)	0.617(±1.337)	0.028	0.129	0.023	0.636
CXCL-10 (IP-10)	1.182(±0.051)	3.400(±0.510)	3.830(±1.000)	<0.0001	<0.0001	<0.0001	0.401
CXCL-2 (MIP-2)	1.304(±0.136)	1.074(±0.048)	1.347(±0.314)	0.027	0.125	0.920	0.028
CCL-2 (MCP-1)	2.095(±0.279)	1.938(±0.294)	1.451(±0.578)	0.015	0.771	0.022	0.056
CCL-3 (MIP-1a)	2.000(±0.610)	1.436(±0.270)	1.227(±0.156)	0.001	0.015	0.001	0.406
CCL-4 (MIP-1b)	2.033(±0.000)	1.234(±0.000)	1.589(±0.000)	0.003	0.002	0.084	0.129
CCL-5 (RANTES)	1.050(±0.004)	1.225(±0.261)	1.417(±0.233)	0.011	0.297	0.009	0.155
CCL-7 (MCP-3)	2.095(±0.279)	2.038(±0.421)	1.465(±0.596)	0.020	0.972	0.045	0.040
Eotaxin (CCL-11)	-1.635(±0.097)	1.101(±1.021)	-0.145(±1.621)	0.001	0.001	0.064	0.085

Supporting Table 4. Intrahepatic gene expression in Mdr2 -/- mice with progressive livery injury and HCC

Data is shown as Log2 fold change (log2FC) from baseline/WT time point. *P* values calculated by one-way ANOVA test for 3 group comparison and Tukey's post hoc test for 2 group comparisons. *P* < 0.05 considered statistically significant.

Intrahepatic genes (Log2 Fold Change from Baseline/WT)	Inflammation/Mdr2-/- Mean (±SD)	Cirrhosis/Mdr2-/- Mean (±SD)	Advanced Cirrhosis/Mdr2-/- Mean (±SD)	HCC/Mdr2-/- Mean (±SD)	*P*-value (one-way ANOVA)	*P*-value Inflammation/Mdr2-/- vs Cirrhosis/Mdr2-/-	*P*-value Inflammation/Mdr2-/- vs Advanced Cirrhosis/Mdr2-/-	*P*-value Inflammation/Mdr2-/- vs HCC/Mdr2-/-	*P*-value Cirrhosis/Mdr2-/- vs Advanced Cirrhosis/Mdr2-/-	*P*-value Cirrhosis/Mdr2-/- vs HCC/Mdr2-/-	*P*-value Advanced Cirrhosis/Mdr2-/- vs HCC/Mdr2-/-
**Innate response**											
Apcs	1.470(±1.070)	1.500(±0.410)	-1.170(±0.890)	-1.170(±2.680)	0.0194	>0.9999	0.0576	0.1198	0.0549	0.1145	0.971
Casp1	2.870(±0.650)	4.420(±0.800)	5.330(±0.600)	4.890(±1.620)	0.0239	0.1842	0.0213	0.0624	0.592	0.9097	0.9241
Cd14	1.470(±1.070)	1.500(±0.410)	3.220(±0.890)	2.490(±0.620)	0.023	>0.9999	0.0373	0.3076	0.041	0.331	0.575
Crp	0.450(±0.400)	0.670(±0.470)	1.020(±0.250)	-1.500(±0.360)	<0.0001	0.843	0.1987	<0.0001	0.5755	<0.0001	<0.0001
Csf2	1.200(±1.550)	2.130(±1.010)	-1.530(±0.390)	-1.680(±1.100)	0.0005	0.6369	0.0188	0.0134	0.0024	0.0017	0.9973
Cxcl10	1.490(±1.070)	1.510(±0.420)	1.740(±0.890)	1.070(±0.680)	0.6997	>0.9999	0.9702	0.879	0.9765	0.864	0.6495
Ddx58	2.310(±0.280)	2.320(±0.380)	1.010(±0.100)	-1.030(±0.670)	<0.0001	>0.9999	0.0038	<0.0001	0.0036	<0.0001	<0.0001
Icam1	2.500(±0.690)	2.990(±0.340)	2.730(±0.450)	1.950(±0.290)	0.0471	0.4787	0.8974	0.3846	0.86	0.0372	0.14
Il1a	1.060(±0.650)	0.780(±0.460)	-1.160(±0.580)	-1.630(±1.000)	0.0002	0.9408	0.0037	0.0008	0.0097	0.0019	0.7808
Il1b	4.580(±1.070)	3.210(±0.660)	1.600(±0.680)	-1.070(±0.840)	<0.0001	0.1438	0.0013	<0.0001	0.0731	<0.0001	0.0032
Il1r1	0.670(±0.450)	1.460(±0.260)	1.450(±0.660)	1.110(±0.780)	0.2222	0.2596	0.2689	0.7044	>0.9999	0.8232	0.8351
Il4	1.450(±1.070)	1.490(±0.410)	-1.640(±0.890)	-1.880(±0.720)	<0.0001	0.9999	0.0008	0.0004	0.0007	0.0004	0.9741
Irak1	0.720(±0.440)	1.080(±0.670)	1.010(±0.400)	-1.040(±0.260)	<0.0001	0.701	0.8154	0.0009	0.9964	0.0002	0.0002
Irf3	1.290(±0.180)	1.170(±0.210)	-1.340(±0.300)	-1.590(±0.500)	<0.0001	0.9512	<0.0001	<0.0001	<0.0001	<0.0001	0.6987
Irf7	1.780(±0.540)	1.610(±0.760)	1.720(±0.440)	1.700(±0.440)	0.9783	0.9724	0.9987	0.9969	0.9921	0.9956	>0.9999
Itgam	4.120(±1.030)	2.140(±0.710)	2.990(±0.740)	-1.010(±1.030)	<0.0001	0.0369	0.3224	<0.0001	0.5514	0.0015	0.0002
Ly96	1.890(±0.460)	2.090(±0.420)	1.650(±0.330)	1.160(±0.600)	0.0727	0.9266	0.8819	0.1701	0.5544	0.0622	0.4687
Lyz2	4.280(±0.600)	6.420(±0.250)	2.400(±0.360)	-1.070(±1.390)	<0.0001	0.0109	0.0247	<0.0001	<0.0001	<0.0001	0.0002
Mapk1	0.900(±0.310)	0.670(±0.250)	-1.210(±0.330)	-1.380(±0.260)	<0.0001	0.6826	<0.0001	<0.0001	<0.0001	<0.0001	0.839
Mapk8	1.810(±0.490)	1.450(±0.650)	-1.380(±0.360)	-1.390(±0.840)	<0.0001	0.8384	<0.0001	<0.0001	0.0001	0.0001	>0.9999
Mbl2	0.580(±0.680)	0.480(±0.480)	-1.340(±0.470)	-1.720(±0.610)	<0.0001	0.9943	0.0022	0.0005	0.0033	0.0007	0.7803
Mx1	1.460(±1.070)	1.480(±0.410)	-1.650(±0.890)	-1.880(±0.720)	<0.0001	>0.9999	0.0008	0.0004	0.0007	0.0004	0.9771
Myd88	1.470(±1.070)	1.500(±0.410)	-1.620(±0.890)	-1.800(±0.650)	<0.0001	>0.9999	0.0007	0.0004	0.0006	0.0004	0.9881
Nfkb1	1.330(±0.190)	1.340(±0.400)	1.030(±0.420)	-1.090(±0.440)	<0.0001	>0.9999	0.6803	<0.0001	0.6585	<0.0001	<0.0001
Nfkbia	1.670(±0.450)	0.780(±0.080)	-1.020(±0.240)	-1.430(±0.230)	<0.0001	0.0038	<0.0001	<0.0001	<0.0001	<0.0001	0.2234
Nlrp3	1.460(±1.070)	1.490(±0.410)	-1.650(±0.890)	-1.880(±0.720)	<0.0001	>0.9999	0.0008	0.0004	0.0007	0.0004	0.9771
Nod1	2.570(±0.700)	1.250(±0.410)	-1.040(±0.340)	-1.240(±0.600)	<0.0001	0.0196	<0.0001	<0.0001	0.0003	0.0001	0.9498
Nod2	1.450(±1.050)	1.400(±0.410)	-1.540(±0.790)	-1.410(±0.580)	<0.0001	0.9997	0.0005	0.0008	0.0006	0.0009	0.9945
Slc11a1	1.460(±1.070)	1.470(±0.410)	-1.180(±0.870)	-1.260(±0.720)	0.0002	>0.9999	0.0028	0.0022	0.0027	0.0021	0.9989
Stat1	1.440(±0.460)	1.700(±0.190)	1.010(±0.140)	-1.080(±0.710)	<0.0001	0.8359	0.5313	<0.0001	0.1724	<0.0001	0.0001
Stat3	0.650(±0.300)	0.560(±0.100)	1.100(±0.380)	-1.130(±0.850)	0.0002	0.9936	0.5833	0.0012	0.4387	0.0019	0.0002
Stat4	0.340(±1.610)	0.420(±1.180)	1.190(±1.140)	-1.120(±0.760)	0.1057	0.9997	0.7561	0.3626	0.8053	0.3202	0.0793
Stat6	1.460(±1.070)	1.360(±0.410)	-1.170(±0.740)	-1.280(±0.370)	<0.0001	0.997	0.001	0.0007	0.0014	0.001	0.996
Ticam1	0.640(±0.540)	0.590(±0.510)	-1.070(±0.820)	1.230(±0.680)	0.0019	0.9995	0.0134	0.5888	0.0162	0.5261	0.0015
Tlr1	3.440(±1.090)	3.940(±0.470)	3.670(±0.770)	3.860(±1.720)	0.1599	0.9187	0.9909	0.9492	0.9855	0.9996	0.9948
Tlr2	3.070(±1.070)	2.950(±0.230)	3.820(±0.850)	2.920(±0.810)	0.379	0.9965	0.5675	0.9932	0.4494	>0.9999	0.4217
Tlr4	1.960(±0.910)	1.760(±0.660)	1.250(±0.620)	-1.090(±0.740)	0.0003	0.9802	0.5481	0.0004	0.7667	0.0007	0.0037
Tlr5	1.050(±0.130)	0.600(±0.410)	-4.510(±0.410)	-2.940(±0.890)	<0.0001	0.6445	<0.0001	<0.0001	<0.0001	<0.0001	0.0064
Tlr6	1.470(±1.070)	1.380(±0.430)	-1.180(±0.810)	1.440(±0.520)	0.0006	0.9982	0.0015	>0.9999	0.002	0.9995	0.0017
Tnf-α	1.470(±0.060)	1.500(±0.410)	-1.640(±0.900)	-1.880(±0.720)	<0.0001	0.9999	<0.0001	<0.0001	<0.0001	<0.0001	0.9436
Tyk2	1.920(±0.400)	1.100(±0.160)	-1.470(±0.510)	-1.110(±0.450)	<0.0001	0.0582	<0.0001	<0.0001	<0.0001	<0.0001	0.6006
**Adaptive response**											
Cd4	2.740(±0.870)	1.330(±0.600)	-1.820(±0.570)	-1.670(±0.790)	<0.0001	0.0698	<0.0001	<0.0001	0.0002	0.0004	0.9906
Cd80	1.510(±1.070)	1.320(±0.420)	-1.250(±0.860)	-2.050(±0.780)	<0.0001	0.9871	0.0022	0.0002	0.0038	0.0004	0.5312
Cd86	2.040(±1.070)	1.440(±0.080)	-1.560(±0.950)	-1.580(±0.460)	<0.0001	0.6804	0.0001	<0.0001	0.0005	0.0005	>0.9999
Cd8a	1.590(±0.670)	1.450(±0.380)	1.500(±0.310)	-1.020(±0.950)	0.0002	0.9887	0.9969	0.0004	0.9995	0.0006	0.0005
Fasl	1.430(±1.040)	1.500(±0.410)	-1.280(±0.890)	-1.370(±0.850)	0.0002	0.9994	0.0029	0.0022	0.0024	0.0018	0.9986
Foxp3	1.420(±1.070)	1.420(±0.410)	-1.720(±0.830)	-1.210(±0.790)	0.0001	>0.9999	0.0007	0.003	0.0007	0.003	0.8101
Ifna2	1.550(±1.070)	1.550(±0.490)	-1.550(±0.820)	-1.770(±0.710)	<0.0001	>0.9999	0.0007	0.0004	0.0007	0.0004	0.9791
Ifnar1	2.020(±0.250)	0.770(±0.360)	-1.300(±0.530)	-1.300(±0.390)	<0.0001	0.0037	<0.0001	<0.0001	<0.0001	<0.0001	>0.9999
Ifnb1	1.460(±1.070)	1.540(±0.460)	-1.840(±0.880)	-1.720(±0.730)	<0.0001	0.999	0.0005	0.0007	0.0004	0.0005	0.9966
Ifng	1.420(±1.070)	1.490(±0.400)	1.640(±0.810)	1.880(±0.720)	0.8477	0.9992	0.9781	0.841	0.9928	0.8949	0.972
Ifngr1	2.160(±0.560)	2.010(±0.060)	2.010(±0.260)	2.290(±0.950)	0.8761	0.9813	0.9813	0.9876	>0.9999	0.8959	0.8959
Il2	0.850(±1.880)	1.600(±0.430)	-1.480(±0.970)	-1.650(±0.980)	0.004	0.8077	0.0691	0.0488	0.0146	0.0102	0.9969
Rag1	1.440(±1.070)	1.550(±0.340)	-1.240(±0.880)	-1.320(±0.710)	0.0002	0.9972	0.0023	0.0018	0.0016	0.0013	0.9989
Rorc	1.840(±0.370)	0.390(±0.850)	-1.540(±0.510)	-2.160(±1.220)	<0.0001	0.1035	0.0004	<0.0001	0.0244	0.0037	0.7043
**Innate and Adaptive response**											
C3	0.920(±0.220)	0.960(±0.500)	1.470(±0.330)	1.120(±0.540)	0.2812	0.9991	0.2942	0.9039	0.3533	0.9471	0.6476
C5ar1	1.500(±1.060)	1.250(±0.120)	1.720(±0.470)	-1.090(±0.850)	0.0004	0.9598	0.9719	0.0013	0.7941	0.003	0.0007
Ccl12	1.390(±1.110)	1.470(±0.460)	-1.750(±0.910)	-1.880(±0.720)	<0.0001	0.9991	0.0009	0.0006	0.0007	0.0005	0.996
Ccl5	1.430(±1.130)	1.310(±0.440)	1.210(±0.680)	-1.080(±0.900)	0.0028	0.9968	0.9811	0.005	0.9981	0.0072	0.0096
Ccr4	1.580(±0.890)	1.650(±0.450)	-1.610(±0.920)	-1.920(±0.260)	<0.0001	0.9989	0.0001	<0.0001	0.0001	<0.0001	0.9188

Ccr5	1.350(±1.070)	1.670(±0.640)	2.070(±0.640)	-1.060(±0.880)	0.0007	0.9456	0.6205	0.0067	0.9013	0.0026	0.0009
Ccr6	1.520(±1.020)	1.450(±0.410)	-1.310(±0.790)	-1.490(±0.610)	<0.0001	0.9991	0.0008	0.0005	0.001	0.0006	0.9855
Ccr8	1.550(±1.060)	1.390(±0.570)	-1.750(±0.980)	-1.480(±0.560)	<0.0001	0.9924	0.0005	0.0011	0.0008	0.0017	0.9658
Cd40	1.410(±1.160)	1.620(±0.430)	-1.620(±0.860)	-1.480(±0.510)	<0.0001	0.9814	0.0008	0.0012	0.0005	0.0007	0.9943
Cd40lg	1.370(±1.090)	1.370(±0.320)	-1.680(±0.950)	-2.170(±0.550)	<0.0001	>0.9999	0.0007	0.0002	0.0007	0.0002	0.8164
Cxcr3	1.450(±1.040)	1.470(±0.380)	-1.480(±0.810)	-1.710(±0.600)	<0.0001	>0.9999	0.0006	0.0003	0.0006	0.0003	0.9714
Gata3	1.460(±0.940)	1.490(±0.510)	-1.660(±0.720)	-1.740(±0.700)	<0.0001	>0.9999	0.0003	0.0002	0.0003	0.0002	0.9986
H2-Q10	0.830(±0.150)	0.630(±0.930)	-1.500(±0.280)	-2.020(±0.470)	<0.0001	0.9528	0.0003	<0.0001	0.0006	<0.0001	0.5511
H2-T23	1.130(±0.330)	0.790(±0.580)	1.480(±0.310)	1.310(±0.490)	0.2036	0.7032	0.6849	0.9374	0.1763	0.3827	0.9464
Il10	1.430(±1.070)	1.530(±0.470)	-1.720(±0.870)	-1.350(±0.720)	<0.0001	0.998	0.0007	0.002	0.0005	0.0015	0.9156
Il13	1.540(±1.040)	1.560(±0.370)	-1.510(±0.820)	-2.050(±0.720)	<0.0001	>0.9999	0.0006	0.0001	0.0006	0.0001	0.761
Il17a	1.470(±1.130)	1.390(±0.330)	-1.000(±0.190)	-1.860(±0.700)	<0.0001	0.9984	0.0014	<0.0001	0.0018	0.0001	0.3379
Il18	1.430(±0.540)	0.930(±0.850)	-1.790(±0.350)	-1.870(±0.640)	<0.0001	0.6748	<0.0001	<0.0001	0.0002	0.0002	0.9977
Il23a	0.960(±1.590)	1.450(±0.530)	-1.260(±0.840)	-1.140(±0.710)	0.0037	0.8984	0.0377	0.0505	0.0112	0.0151	0.9982
Il5	1.360(±1.010)	1.360(±0.220)	-1.160(±0.880)	-1.980(±0.710)	<0.0001	>0.9999	0.0027	0.0002	0.0027	0.0002	0.4597
Il6	1.510(±1.070)	1.590(±0.450)	-1.430(±0.970)	-2.230(±0.350)	<0.0001	0.9988	0.0009	<0.0001	0.0007	<0.0001	0.4905
Jak2	1.430(±0.400)	1.140(±0.530)	-1.020(±0.400)	-1.180(±0.280)	<0.0001	0.7549	<0.0001	<0.0001	<0.0001	<0.0001	0.945
Mpo	1.450(±1.160)	1.550(±0.390)	2.900(±0.890)	1.440(±1.190)	0.1425	0.9988	0.1985	>0.9999	0.247	0.9984	0.1941
Tbx21	1.600(±1.150)	1.430(±0.470)	-1.630(±0.870)	-1.970(±0.710)	<0.0001	0.9913	0.0007	0.0003	0.0012	0.0005	0.9379
Tlr3	2.650(±0.440)	2.430(±0.800)	-1.190(±1.010)	-1.340(±0.660)	<0.0001	0.9755	<0.0001	<0.0001	0.0001	<0.0001	0.9919
Tlr7	1.240(±1.620)	1.360(±0.330)	-1.670(±0.750)	-1.610(±0.580)	0.0005	0.9979	0.0048	0.0056	0.0035	0.0041	0.9997
Tlr8	1.860(±1.070)	1.270(±0.700)	1.340(±0.500)	-1.320(±0.210)	0.0001	0.6375	0.7196	0.0002	0.9989	0.001	0.0008
Tlr9	1.490(±1.120)	1.360(±0.420)	-1.680(±0.780)	-2.620(±0.590)	<0.0001	0.995	0.0004	<0.0001	0.0006	<0.0001	0.3555
Traf6	2.160(±0.780)	0.920(±0.680)	-1.770(±0.300)	-1.440(±0.690)	<0.0001	0.0738	<0.0001	<0.0001	0.0003	0.0011	0.8834
